# miR-424-5p Regulates Stem-like and Malignant Phenotypes in Osteosarcoma by Targeting FZD4

**DOI:** 10.3390/cancers18172846

**Published:** 2026-09-03

**Authors:** Jingjin Ma, Yi Yang, Tong Liu, Jiaxing Chen, Zhiyu Chen, Yunsheng Jiang, Xinyu Yang, Junhong Chen, Xu Zhou, Tao He, Zhengxue Quan

**Affiliations:** 1 Department of Orthopaedic Surgery, The First Affiliated Hospital of Chongqing Medical University, Chongqing 400016, China; 2 A Campus Clinic, Hospital of Chongqing University, Chongqing 400044, China; 3 Department of Orthopaedic Trauma, Chongqing General Hospital, Chongqing University, Chongqing 401147, China

**Keywords:** OS, FZD4, stemness, microRNA, tumor microenvironment

## Abstract

**Background/Objectives:** OS is characterized by marked tumor heterogeneity, stemness-associated phenotypes, metastatic potential, and poor prognosis. FZD4 is an important Wnt signaling receptor involved in stem cell regulation and tumor progression, but its role and microRNA-mediated regulation in OS remain unclear. The aim of this study was to investigate the relationship between FZD4 expression, OS stemness, malignant progression, and upstream microRNA regulation. **Methods:** *Human* OS single-cell RNA sequencing data were analyzed using Seurat, CytoTRACE, and Monocle2. Tumor cell subpopulations, developmental potential, and pseudotime trajectories were evaluated. Functional enrichment analyses were performed to identify pathways associated with high FZD4 expression. MicroRNA–mRNA interaction analysis was used to predict upstream regulatory microRNAs, and immunohistochemistry, Western blotting, and in vitro and in vivo experiments were performed for validation. **Results:** FZD4 was mainly enriched in OS tumor cell subpopulations and was significantly associated with poor prognosis. Tumor cells with high FZD4 expression showed increased developmental potential, stemness features, and enrichment in early developmental states. Functional enrichment analysis indicated activation of epithelial–mesenchymal transition, angiogenesis, inflammatory response, KRAS signaling, hypoxia, glycolysis, and Wnt-related pathways. Clinical validation confirmed elevated expression of FZD4, OCT4, and SOX2 in OS tissues. FZD4 knockdown inhibited OS cell stem-like phenotypes and malignant behaviors, proliferation, and migration, while *miR-424-5p* suppressed these malignant phenotypes by targeting *FZD4*. Importantly, rescue experiments demonstrated that restoration of FZD4 substantially reversed the inhibitory effects of *miR-424-5p* mimics on sphere formation, migration/invasion, proliferation, and stemness-associated cell populations. **Conclusions:** Our findings identify *miR-424-5p* as an upstream suppressor of FZD4 and demonstrate that the *miR-424-5p/FZD4* axis regulates stem-like and malignant phenotypes of OS cells, at least partly through Wnt/β-catenin signaling. This axis may represent a potential therapeutic target for OS.

## Simple Summary

Osteosarcoma (OS) is an aggressive bone cancer with high diversity among tumor cells and poor outcomes for many patients. The factors that drive tumor growth, spread, and treatment resistance must be better understood to identify new biomarkers and therapeutic targets. In this study, the authors investigated the role of frizzled class receptor 4 in OS progression, especially its relationship with cancer cell stem-like properties and migration. Using single-cell analysis, clinical samples, and laboratory experiments, they found that frizzled class receptor 4 (FZD4) is highly expressed in OS and is associated with poor prognosis. Reducing its expression weakened the stem-like characteristics and migration ability of OS cells. This study also shows that *miR-424-5p* can regulate these tumor-promoting effects by targeting *FZD4*. These findings may help researchers develop new strategies for predicting prognosis and treating OS.

## 1. Introduction

Osteosarcoma (OS) is the most common primary malignant bone tumor affecting adolescents and young adults, characterized by aggressive behavior, a high propensity for early metastasis, and an unfavorable prognosis [1,2]. Despite advances in chemotherapy, targeted therapy, and immunotherapy, the prognosis of recurrent and metastatic OS remains unsatisfactory. Therefore, identifying novel molecular mechanisms involved in OS progression is essential for improving patient outcomes [3,4,5].

Tumor heterogeneity and cancer stemness are increasingly recognized as key factors contributing to OS progression, metastasis, and therapeutic resistance [6,7]. Cancer stem-like cells possess strong self-renewal capacity and an undifferentiated phenotype, thereby promoting tumor recurrence and treatment failure [8,9,10]. Recent advances in single-cell transcriptomic sequencing have provided powerful tools for exploring tumor heterogeneity and identifying stemness-associated tumor cell subpopulations [11,12,13].

MicroRNAs are short non-coding RNAs that regulate gene expression at the post-transcriptional level and participate in various biological processes, including proliferation, apoptosis, stemness, and tumor progression [14,15,16]. Increasing evidence indicates that dysregulated microRNAs promote OS progression by modulating oncogenic signaling pathways and stemness-related programs [17]. However, the relationship between microRNA-mediated regulation and FZD4 expression in OS remains unclear.

The Wnt signaling pathway is an important regulator of embryonic development, stem cell maintenance, and tumor progression [18,19]. Frizzled receptors are key receptors for Wnt ligands and regulate multiple downstream signaling pathways involved in proliferation, migration, differentiation, and stemness [20,21]. FZD4 is associated with angiogenesis and tumor progression in various cancers [22,23,24,25]. However, the biological role of FZD4 in OS and its upstream microRNA-mediated regulatory mechanism remain poorly understood.

In this study, we investigated the role of FZD4 in OS by integrating single-cell transcriptomic analysis, CytoTRACE analysis, pseudotime trajectory analysis, microRNA regulatory network analysis, and validation using clinical samples as well as in vitro and in vivo experiments. Our findings suggest that FZD4 is associated with stemness-related features, OS migration, and a microRNA-mediated regulatory network in OS.

## 2. Materials and Methods

### 2.1. Experimental Design, Data Acquisition, and Cellular Landscape of OS

The GSE152048 dataset, comprising samples from 11 patients with OS, was obtained from the Gene Expression Omnibus (GEO). In addition, mRNA sequencing data and corresponding clinical information for 88 OS patients were collected from the UCSC Xena database. All datasets used in this study were derived from publicly available sources or previously published research; therefore, no ethical approval was required for their use.

scRNA-seq data were processed using the Seurat package (version 4.4.0) in R (version 4.3.3). Quality control thresholds were set as follows: nFeature_RNA > 200, nFeature_RNA < 4000, and percent.mt < 10. Subsequently, the “LogNormalize” method was applied for normalization with a scale factor of 10,000. The top 2000 most variable genes were selected, followed by batch effect correction using Harmony (version 1.2.0). Dimensionality reduction and visualization were performed using t-SNE and UMAP. Cell clusters were annotated based on the expression of canonical marker genes, with annotation and visualization carried out using functions implemented in the Seurat package (version 4.4.0).

### 2.2. Cell Annotation and Cluster Identification

Classical marker genes were used to identify distinct cell populations, as previously described [26,27]. Specifically, OS cells were identified by *ALPL*, *COL1A1, RUNX2*, and *IBSP*; B cells by *JCHAIN, MZB1, ICHG1*, and *IGKC*; endothelial cells by *VWF* and *PECAM1*; osteoclasts by *CTSK, MMP9*, and *ACP5; NK/T* cells by *IL7R, CD3D, NKG7*, and *CD247*; myeloid cells by *IL1B, NLRP3, MS4A7*, and *IL1RN*; fibroblasts by *ASPN*, *ITGBL1, FNDC1*, and *CXCL14*; and pericytes by *ACTA2* and *RGS5*.

To further distinguish malignant OS cells from nonmalignant osteogenic populations, inferCNV analysis was performed to infer large-scale copy-number alterations from single-cell transcriptomic profiles. Cells exhibiting tumor-associated copy-number alteration patterns were classified as malignant OS cells and extracted for secondary clustering and subsequent analyses.

### 2.3. Pseudotime Trajectory Analysis of OS Cells

We applied the Monocle2 R package (version 2.28.0) to analyze the developmental progression of OS subtypes, investigate pseudotime trajectories, and examine the dynamics of mitophagy regulators. Before this, we used the cytotrace2 package (version 1.1.0) to predict cell stemness. Furthermore, the GSVA package (version 1.48.3) was employed to examine the expression correlations among OS subtypes and gene set enrichment. Highly variable genes were selected based on the following criteria: mean expression ≥ 0.1 and empirical dispersion ≥ 0.1 × dispersion fit.

The plot_pseudotime_heatmap function was then used to generate heatmaps illustrating the temporal expression patterns of pseudo-related genes across different OS subtypes along the inferred pseudotime trajectory. Malignant OS cells identified by inferCNV were used for pseudotime trajectory analysis. The root state of the Monocle2 trajectory was defined by integrating CytoTRACE2-derived developmental potential with stemness-associated scores. Cell populations exhibiting relatively higher developmental potential and stemness-associated characteristics were assigned as the starting population of the inferred trajectory.

### 2.4. Cell Enrichment Analysis

To evaluate transcriptional differences associated with *FZD4* expression, malignant OS cells were stratified into *FZD4*-high and *FZD4*-low groups according to the median *FZD4* expression level across the malignant OS cell population. The median expression value of 0.2766269 was used as the cutoff, with cells above this value classified as *FZD4*-high and those at or below this value classified as *FZD4*-low. Differential gene expression analysis was subsequently performed between the two groups.

Survival analyses were performed using the TARGET-OS cohort as the training cohort (n = 88), while GSE21257 and GSE39055 were combined as an independent validation cohort (n = 90). Kaplan–Meier survival curves were compared using the log-rank test. The association between *FZD4* expression and overall survival was additionally estimated using Cox proportional-hazards analysis and reported as the hazard ratio (HR) with 95% confidence interval (CI).

### 2.5. Cell–Cell Communication Analysis

We employed the CellChat package (version 1.6.1) to analyze cell–cell interactions following the standard pipeline to investigate potential intercellular communication networks. Ligand–receptor pairs involved in secreted signaling were extracted from the CellChatDB database (human) and used to infer communication patterns among distinct cell populations. Cell groups containing fewer than 10 cells were excluded, and only communications with a significance threshold of ≤0.05 were considered meaningful. These ligand–receptor interactions were subsequently integrated into protein–protein interaction (PPI) networks, enabling the quantification of interaction strength based on computed probabilities of cell–cell communication. Using this framework, we systematically characterized molecular interaction networks involving key cell types (*FZD4*-high and *FZD4*-low OS cells), with a particular focus on communication pathways involving other cell types.

### 2.6. miRNA–mRNA Regulatory Network Analysis

The starBase prediction database, together with transcriptomic sequencing results, was used to predict potential upstream miRNAs targeting *FZD4* [28]. Correlation analysis between *FZD4* expression and candidate miRNAs was performed to identify potential regulatory interactions associated with OS progression.

### 2.7. Immunohistochemistry and Western Blot Analysis

Human OS tissues and adjacent non-tumor tissues were collected. A total of n = 5 osteosarcoma tissues and n = 4 adjacent normal bone tissues were included for IHC analysis. Immunohistochemical staining was performed using anti-FZD4, anti-OCT4, and anti-SOX2 antibodies. Western blot experiments were performed using three independent biological replicates (n = 3). The corresponding original uncropped Western blot images are provided in Supplementary File S1.

### 2.8. Cell Culture

Human OS cell lines 143B, HOS, MG63, and U2OS were purchased from the Cell Bank of the Chinese Academy of Sciences, Shanghai, China. Cells were cultured in Dulbecco’s modified Eagle’s medium (DMEM; Gibco, Grand Island, NY, USA) supplemented with 10% fetal bovine serum (FBS; Gibco) and 1% penicillin–streptomycin solution under humidified conditions at 37 °C with 5% CO_2_.

HOS cells were used for primary mechanistic experiments due to their highest endogenous *FZD4* expression. Selected validation experiments were additionally performed in 143B cells as an independent osteosarcoma model.

All cell lines used in this study were routinely tested for mycoplasma contamination and authenticated via short tandem repeat (STR) profiling before experiments.

### 2.9. Quantitative Real-Time PCR

Total RNA was extracted using TRIzol reagent (NCM Biotech, Suzhou, China), followed by cDNA synthesis using PrimeScript RT reagent (Thermo Fisher, Waltham, MA, USA). Quantitative amplification was performed in triplicate on a Bio-Rad CFX96 platform using SYBR Select Master Mix (Hercules, CA, USA). The amplification program consisted of an initial denaturation at 95 °C for 30 s, followed by 40 cycles at 95 °C for 5 s and 60 °C for 30 s.

Gene expression levels were normalized using the comparative Ct method, with *GAPDH* serving as the internal control. The primer sequences were as follows: *GAPDH*, forward 5′-GGAGTCCACTGGCGTCTTCA-3′ and reverse 5′-GTCATGAGTCCTTCCACGATACC-3′; *FZD4*, forward 5′-ACCTTGGCTTCCTAAAGTGTTG-3′ and reverse 5′-CTGCCTGAGAGACAGAGTAAGA-3′; *OCT4*, forward 5′-CCTGAAGCAGAAGAGGATCACC-3′ and reverse 5′-AAAGCGGCAGATGGTCGTTTGG-3′; and *SOX2*, forward 5′-TACCTCTTCCTCCCACTCCA-3′ and reverse 5′-GGGCAGTGTGCCGTTAATG-3′.

Quantitative real-time PCR analysis was performed using three independent biological replicates, with each replicate representing samples obtained from independently cultured and treated cells. The data were analyzed using Student’s *t*-test, and *p* < 0.05 was considered statistically significant.

### 2.10. Wound-Healing Assay

OS cells were trypsinized and seeded into 6-well plates at a density of approximately 1 × 10^6^ cells per well, followed by overnight culture, to form a confluent monolayer. A straight scratch was made vertically across the bottom of each well using a sterile 200 μL pipette tip. The wells were gently washed twice with PBS to remove detached cell debris. DMEM containing 1% FBS was then added to suppress cell proliferation, and the cells were cultured at 37 °C with 5% CO_2_.

Images of cell migration were captured under an inverted microscope at 0, 12, and 24 h after scratching. The wound area was measured using the ImageJ software (version 1.51j8), and the wound closure percentage was calculated using the following formula:

Wound closure percentage (%) = (wound area at 0 h − wound area at the indicated time point)/wound area at 0 h × 100%.

Each experiment was performed using three independent biological replicates, with three technical replicate wells per group (n = 3).

### 2.11. Transwell Migration and Invasion Assays

Cell migration and invasion assays were performed using Transwell chambers (Corning, NY, USA). For the invasion assay, the upper chambers were precoated with Matrigel (BD Biosciences, San Jose, CA, USA). OS cells suspended in serum-free medium were seeded into the upper chambers, while complete medium containing 10% FBS was added to the lower chambers. After incubation for 24–48 h, migrated or invaded cells were fixed, stained with crystal violet, and counted under a microscope.

### 2.12. Mouse Xenograft Tumor Model

Female BALB/c nude *mice* aged 4–6 weeks were purchased from Chongqing Huasuo Tongjie Laboratory Animal Sales Co., Ltd., Chongqing, China. All animal experiments were approved by the Institutional Animal Care and Use Committee and conducted in accordance with institutional guidelines.

OS cells were suspended in phosphate-buffered saline and subcutaneously injected into the flanks of nude *mice*. Tumor growth was monitored every three days, and tumor volume was calculated using the following formula:

Volume = 0.5 × length × width^2^.

For the *miR-424-5p* treatment experiment, *mice* bearing established OS xenografts were randomly allocated to the control and *miR-424-5p* mimic groups (n = 5 per group). *miR-424-5p* mimics were administered at 5 nmol/mouse via intratumoral injection, once every 3 days, for 18 days. The control group received an equivalent amount of negative-control mimic using the same formulation and administration schedule.

At the experimental endpoint, the mice were sacrificed, and tumor tissues were collected for immunohistochemistry, Western blot analysis, and transcriptomic analysis. Five mice were randomly assigned to each group (n = 5).

### 2.13. FZD4 Rescue Experiments

Rescue experiments were performed by co-transfecting osteosarcoma cells with *miR-424-5p* mimics and an FZD4 overexpression plasmid to determine whether the biological effects of *miR-424-5p* were mediated through *FZD4*. For the rescue experiments, the *FZD4* coding sequence (CDS), without the endogenous *FZD4* 3′UTR, was cloned into the GV493 lentiviral expression vector (GeneChem Co., Ltd., Shanghai, China). The efficiency of FZD4 overexpression was confirmed using Western blotting. Cells were divided into the control (CON), miR-424-5p mimic (mimics), and *miR-424-5p* mimic plus FZD4 overexpression (mi+Oe-FZD4) groups. Migration/invasion, sphere-forming capacity, cell proliferation, stemness-associated cell populations, and expression of FZD4, β-catenin, OCT4, and SOX2 were subsequently evaluated. All rescue experiments were independently repeated three times (n = 3).

### 2.14. Statistical Analysis

Statistical analyses were performed using R software and GraphPad Prism (version 9.4.0). Kaplan–Meier survival analysis was conducted using the log-rank test. Comparisons between two groups were performed using Student’s *t*-test. Tumor growth curves were analyzed using two-way ANOVA followed by Tukey’s multiple comparisons test. A *p*-value of <0.05 was considered statistically significant.

## 3. Results

### 3.1. Single-Cell Transcriptomic Analysis Reveals OS Cell Heterogeneity and Differences in Developmental Potential

**Figure 1:**
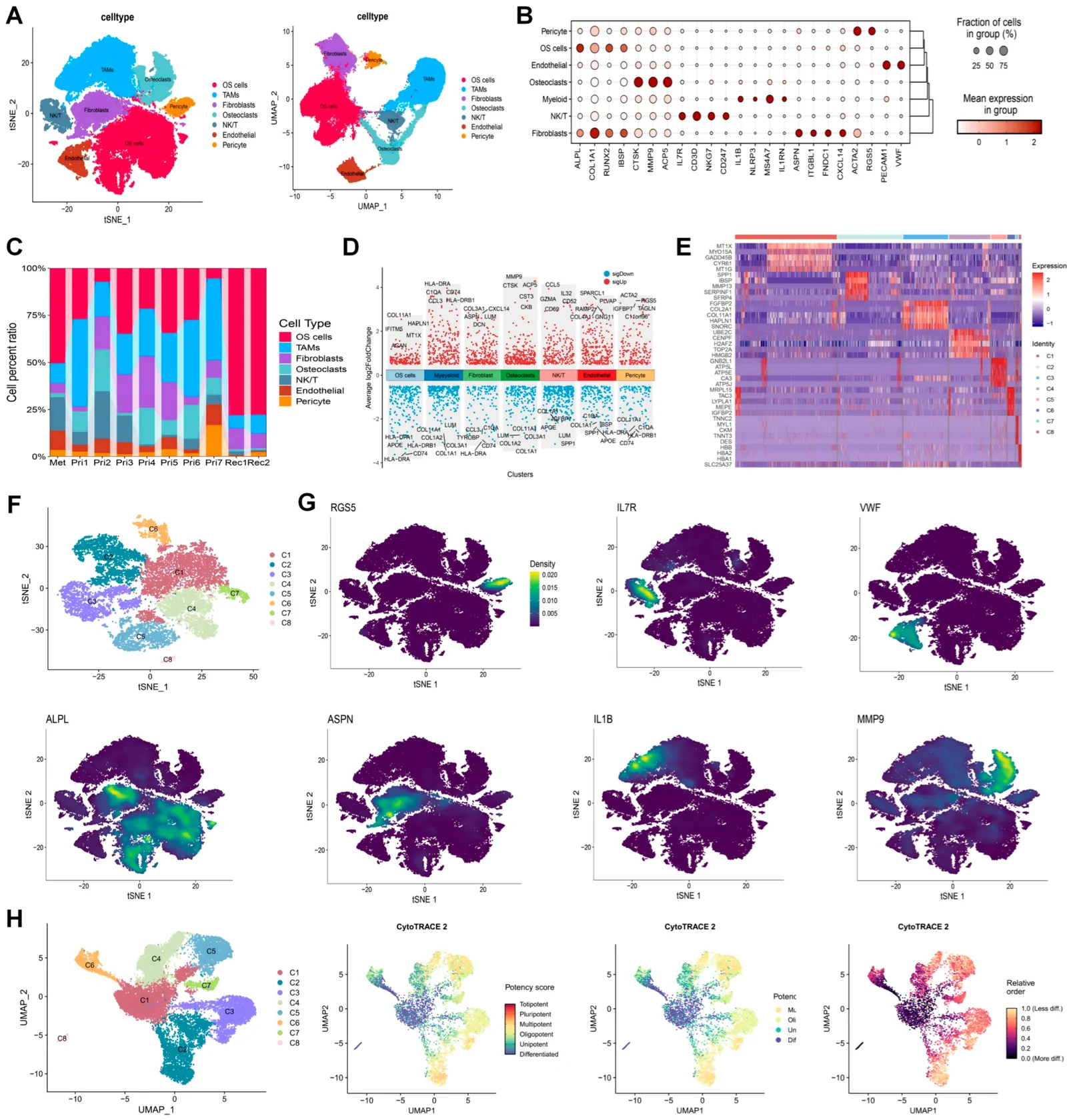
Single-cell transcriptomic analysis reveals cellular heterogeneity and differential developmental potential of osteosarcoma cells. (**A**) t-SNE and UMAP dimensional reduction analyses identifying major cellular populations within the osteosarcoma (OS) tumor microenvironment, including OS cells, tumor-associated macrophages (TAMs), fibroblasts, osteoclasts, NK/T cells, endothelial cells, and pericytes. (**B**) Dot plot showing the expression of representative marker genes in different cell populations. (**C**) Cell composition analysis across different patient samples, demonstrating substantial interpatient heterogeneity within the OS tumor microenvironment. (**D**) Representative marker gene expression patterns among different cell populations. Upregulated genes are shown in red, while downregulated genes are shown in blue. (**E**) Heatmap showing differentially expressed genes among OS cell subclusters (C1–C8) after reclustering analysis. (**F**) t-SNE plot showing eight distinct OS cell subclusters (C1–C8) identified after further reclustering of OS cells. (**G**) Feature plots showing the expression distribution of representative marker genes, including *RGS5, IL7R, VWF, ALPL, ASPN, IL1B*, and *MMP9*, across single cells. (**H**) CytoTRACE2 analysis showing the developmental potential and differentiation states of different OS cell subclusters. The left panel shows subcluster distribution, the middle panels show potency score and potency category, and the right panel shows relative differentiation states.

Single-cell transcriptome sequencing analysis was performed to investigate the cellular heterogeneity and developmental characteristics of OS. t-SNE and UMAP analyses identified multiple cell populations within the OS tumor microenvironment, including OS cells, TAMs, fibroblasts, osteoclasts, NK/T cells, endothelial cells, and pericytes (Figure 1A). Cell identity was determined based on canonical marker genes. Specifically, OS cells exhibited high expression of osteogenic markers *ALPL* and *RUNX2*, myeloid cells were enriched for inflammatory markers *IL1B* and *MMP9*, fibroblasts showed elevated *ASPN* expression, endothelial cells expressed *VWF*, and pericytes were characterized by *RGS5* expression (Figure 1B,G). Cell composition analysis revealed substantial interpatient heterogeneity among different OS samples (Figure 1C). The expression patterns of representative marker genes further demonstrated distinct transcriptional characteristics across different cell populations (Figure 1D). OS cells were extracted and reclustered into eight transcriptionally distinct subclusters to explore intratumoral heterogeneity (C1–C8), indicating remarkable diversity among malignant OS cells (Figure 1E,F). CytoTRACE2 analysis revealed significant differences in developmental potential among OS cell subclusters. C3, C4, and part of the C5/C6 populations exhibited higher potency scores and relatively lower differentiation states, whereas C7 and C8 displayed more differentiated characteristics (Figure 1H). These findings indicate that OS contains distinct tumor cell populations with variable developmental potential and stem-like characteristics, providing a basis for further investigation into molecular regulators associated with OS progression.

### 3.2. FZD4 Is Closely Associated with Developmental Potential and Stemness Features in OS Cells

**Figure 2:**
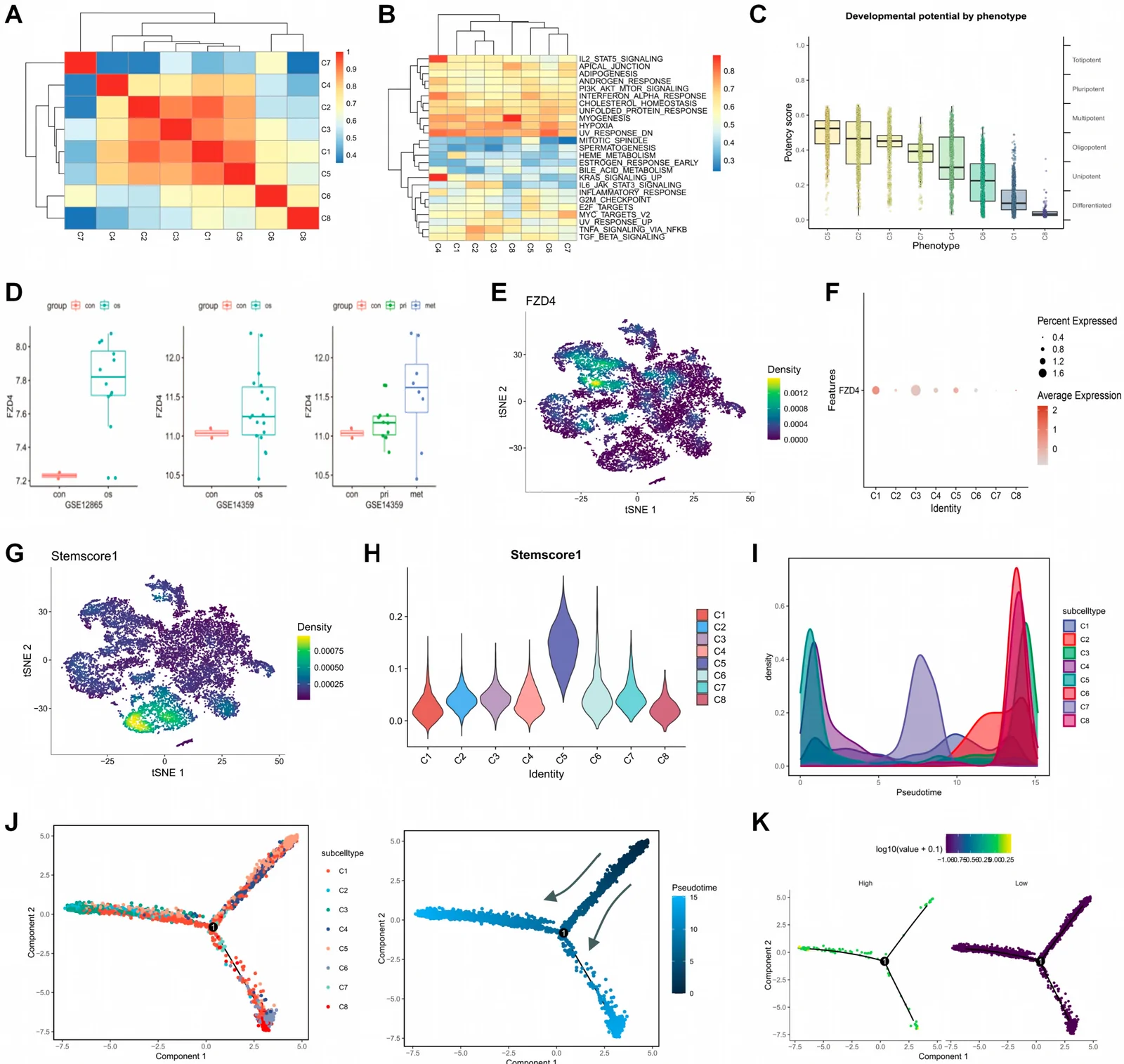
*FZD4* is associated with developmental potential and stem-like characteristics in OS cells. (**A**) Correlation heatmap among OS cell subclusters. (**B**) Hallmark pathway enrichment analysis showing the differential enrichment of biological pathways among OS cell subclusters. (**C**) Analysis of developmental potential, shown as potency scores, across different OS cell subclusters (C1–C8). (**D**) Validation of *FZD4* expression levels in external datasets, GSE126209 and GSE14359, including normal tissues, OS tissues, and metastatic tissues. (**E**) Feature plot and dot plot showing the single-cell expression distribution of *FZD4* and its expression levels across different cell subclusters. (**F**) Dot plot displaying the expression level and percentage of *FZD4*-positive cells among different OS subclusters. Dot size represents the fraction of expressing cells, while color intensity represents the average expression level. (**G**) Stemness-associated score analysis. The left panel shows the distribution of stemness scores at the single-cell level, and the right panel compares stemness scores among different OS cell subclusters. (**H**) Violin plot showing stemness score differences among OS cell subclusters. (**I**) Density plot showing the distribution of different OS cell subclusters along pseudotime. (**J**) Monocle pseudotime trajectory analysis showing different developmental branches and evolutionary directions of OS cells. The left panel shows the distribution of different cell subclusters, and the right panel shows changes in pseudotime states. (**K**) Pseudotime trajectory analysis of *FZD4*-high and *FZD4*-low cells, showing that cells with high *FZD4* expression are mainly enriched in early developmental stages.

Correlation analysis showed that the C1–C4 cell populations exhibited high transcriptomic similarity, whereas C7 and C8 displayed distinct transcriptional profiles, indicating substantial molecular heterogeneity among OS cell subclusters (Figure 2A). Hallmark pathway enrichment analysis revealed that cell populations with high developmental potential were significantly enriched in tumor-promoting pathways, including hypoxia, TNFα signaling via NF-κB, phosphoinositide 3-kinase–protein kinase B–mechanistic target of rapamycin signaling, epithelial–mesenchymal transition, and inflammatory response (Figure 2B). CytoTRACE analysis further showed that C3, C4, and part of the C6 cell population had higher potency scores, whereas C7 and C8 were mainly in a differentiated state (Figure 2C). Validation using online datasets showed that *FZD4* was significantly upregulated in OS tissues and was further increased in metastatic tissues (Figure 2D). Feature plot and dot plot analyses revealed that *FZD4* expression was preferentially enriched in specific OS cell subclusters with higher developmental potential (Figure 2E,F). Stemness-associated score analysis showed that *FZD4*-enriched cell populations exhibited increased stem-like transcriptional characteristics, with C5 displaying the highest stemness score among OS subclusters (Figure 2G,H). Pseudotime trajectory analysis further demonstrated that *FZD4* expression varied along the inferred developmental trajectory, with FZD4-high cells preferentially distributed in less differentiated states, whereas *FZD4*-low cells were enriched in more differentiated regions (Figure 2I–K). Collectively, these findings suggest that *FZD4* is closely associated with maintenance of stem-like properties, progenitor-like phenotype, and malignant progression in OS cells.

### 3.3. FZD4 and Stemness-Related Molecules Are Highly Expressed in OS Tissues

**Figure 3:**
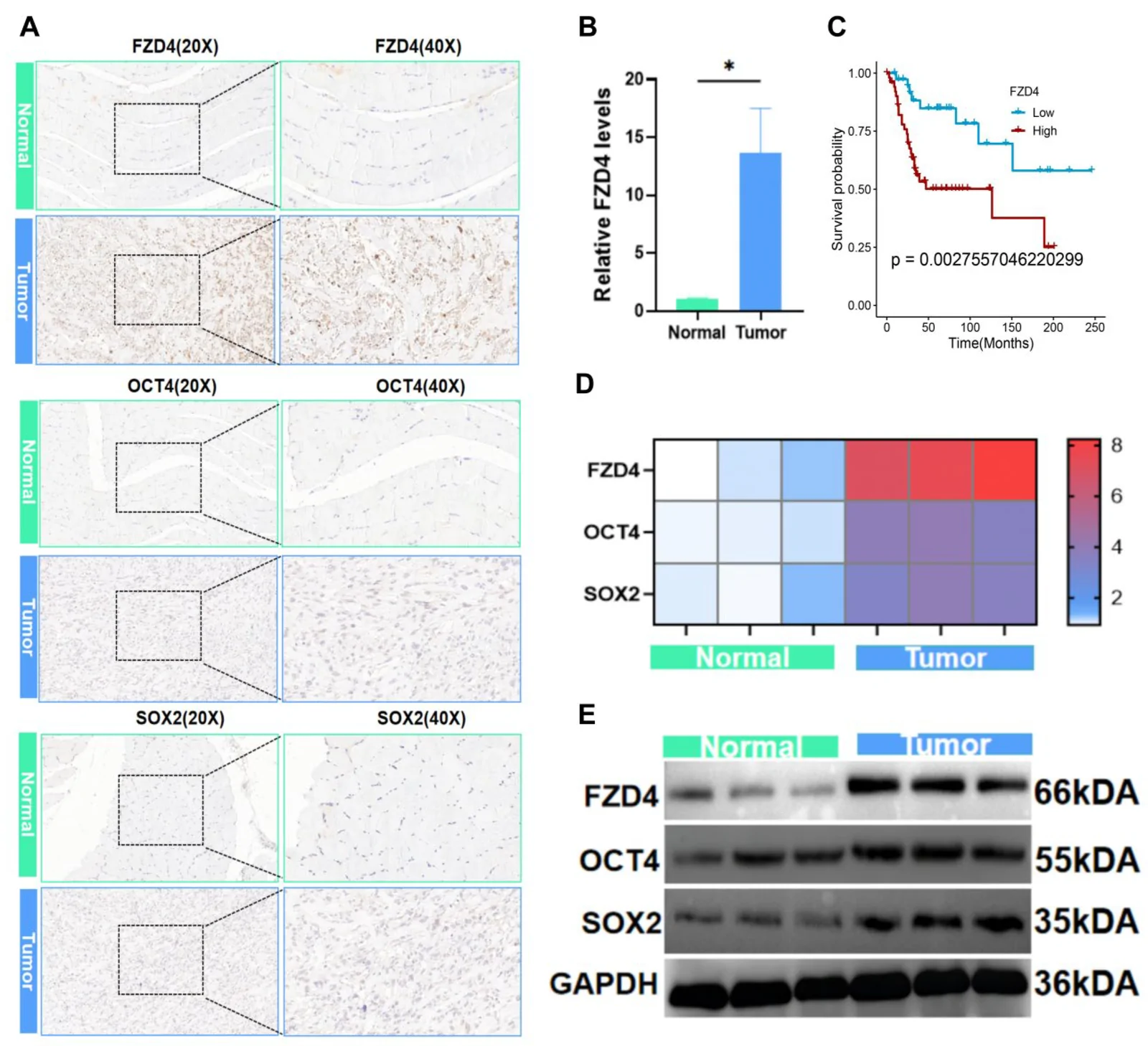
Expression and clinical significance of FZD4 and stemness-related molecules in OS tissues. (**A**) Immunohistochemical staining showing the expression of FZD4, OCT4, and SOX2 in normal bone tissues and OS tissues. (**B**) Statistical analysis of the relative expression levels of FZD4 in normal tissues and OS tissues. (**C**) Kaplan–Meier survival analysis showing that patients with high *FZD4* expression had significantly lower overall survival than those with low FZD4 expression. (**D**) qPCR heatmap showing changes in the overall messenger RNA expression levels of FZD4, OCT4, and SOX2 in normal tissues and OS tissues. (**E**) Western blot analysis of FZD4, OCT4, and SOX2 protein expression levels in normal tissues and OS tissues (Image S1). * *p* < 0.05.

Immunohistochemical staining showed stronger positive signals for FZD4, OCT4, and SOX2 in OS tissues than in normal bone tissues, with markedly enhanced cytoplasmic and nuclear staining observed at 40× magnification (Figure 3A). Statistical analysis supported that FZD4 expression was significantly higher in OS tissues than in normal tissues (Figure 3B). Kaplan–Meier survival analysis indicated an association between higher *FZD4* expression and poorer overall survival (Figure 3C). In the TARGET-OS training cohort (n = 88), the estimated HR for FZD4 was 3.435 (95% CI, 0.151–78.169). Given the very wide confidence interval, this result indicates substantial statistical uncertainty and should be interpreted as an exploratory association rather than evidence that FZD4 is an independent prognostic factor. In addition, the overall messenger RNA expression levels of FZD4, OCT4, and SOX2 were markedly elevated in tumor tissues (Figure 3D). Western blotting further validated the high expression of FZD4, OCT4, and SOX2 in OS tissues (Figure 3E). These results suggest that FZD4 may be closely associated with maintenance of stem-like properties and malignant progression in OS.

### 3.4. FZD4 Promotes Stemness Maintenance and Undifferentiated Phenotype in OS Cells

**Figure 4:**
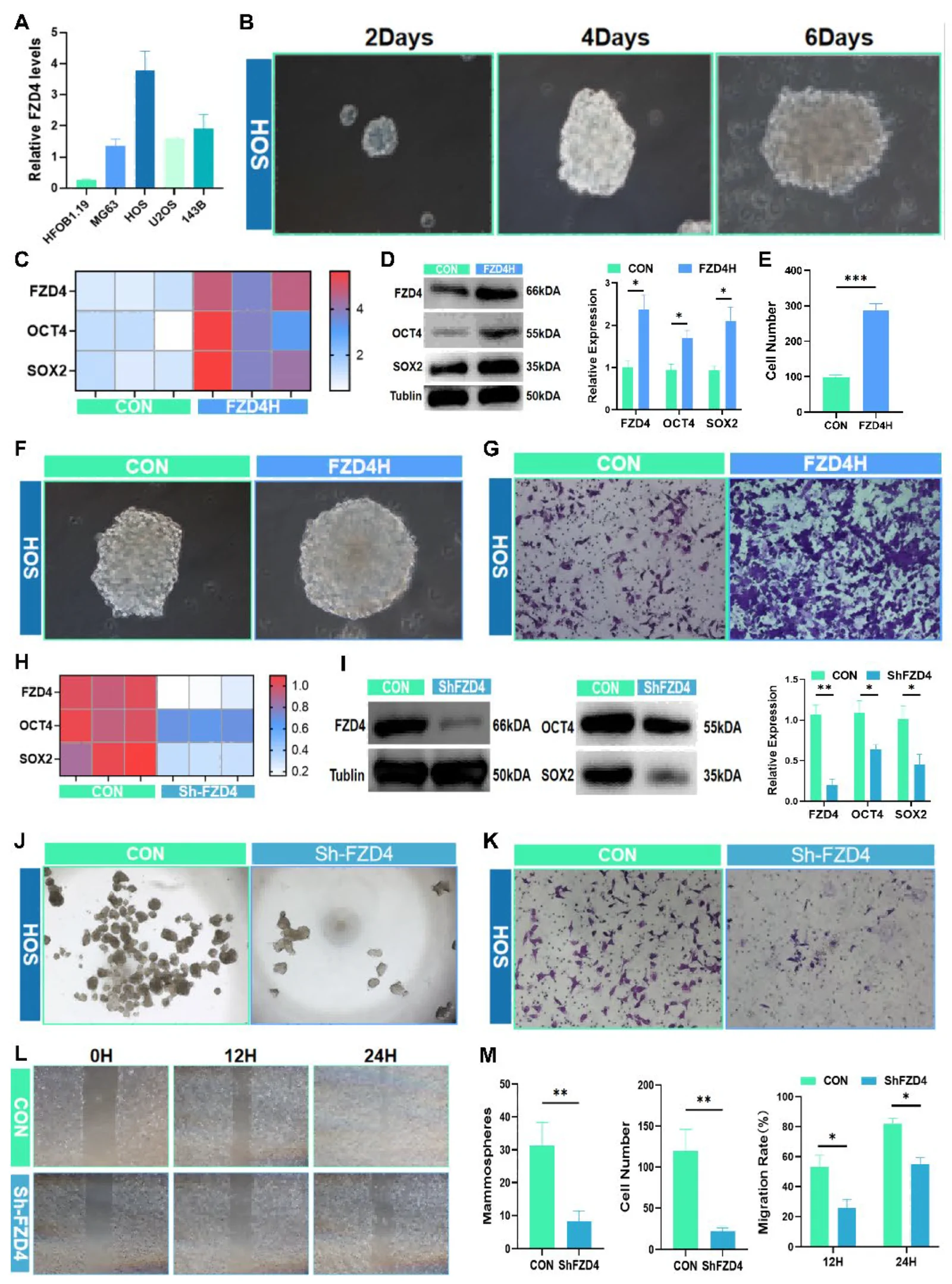
FZD4 promotes stemness maintenance, migration, and invasion in OS cells. (**A**) qPCR analysis of *FZD4* expression levels in different cell lines. (**B**) Sphere-formation assay showing the sphere-forming process of OS cells on days 2, 4, and 6. (**C**) qPCR heatmap showing changes in the expression of *FZD4, OCT4*, and *SOX2* after *FZD4* overexpression. (**D**) Western blot analysis of FZD4, OCT4, and SOX2 protein expression levels after sphere formation (Image S2). (**E**) Statistical analysis of the number of invasive cells after FZD4 overexpression. (**F**) Representative images of sphere formation in the FZD4-overexpression and control groups. (**G**) Transwell assay assessing changes in the migration/invasion ability of OS cells after FZD4 overexpression. (**H**) qPCR heatmap showing changes in the expression of *FZD4, OCT4*, and *SOX2* after *FZD4* knockdown. (**I**) Western blot analysis of FZD4, OCT4, and SOX2 protein expression levels after shFZD4 treatment (Image S3). (**J**) Representative images of the sphere formation assay after FZD4 knockdown. (**K**) Transwell assay assessing changes in the invasive ability of OS cells after FZD4 knockdown. (**L**) Wound-healing assay evaluating the migratory ability of cells in the shFZD4 and control groups at 0, 12, and 24 h. (**M**) Statistical analysis of mammosphere number, number of cells penetrating the Transwell membrane, and wound-healing migration rate. Data are presented as means ± SD. * *p* < 0.05, ** *p* < 0.01, and *** *p* < 0.001.

qPCR results showed that *FZD4* was highly expressed in OS cell lines, with the highest expression observed in HOS cells (Figure 4A); therefore, HOS cells were used for subsequent experiments. The sphere formation assay demonstrated that OS cell spheres gradually increased in size with prolonged culture time (Figure 4B). qPCR and Western blot analyses showed that the expression levels of FZD4, OCT4, and SOX2 were significantly upregulated in enriched cell spheres (Figure 4C,D). Flow cytometry analysis showed that the proportion of stemness-associated cells was significantly increased in the FZD4H group compared with the CON group (Figure S1), accompanied by a marked increase in sphere size and the number of cells penetrating the Transwell membrane (Figure 4E–G, Figures S2 and S3), suggesting that FZD4 enhances OS cell stem-like phenotype and invasive capacity. In contrast, knockdown of FZD4 markedly decreased the expression of OCT4 and SOX2 (Figure 4H,I and Figure S4), significantly impaired sphere-forming ability, and notably reduced Transwell migration/invasion capacity. Wound-healing assays further showed that the migration rate of cells in the shFZD4 group was markedly slowed (Figure 4J–M). These findings indicate that FZD4 promotes maintenance of stem-like properties, migration, and invasion in OS cells.

### 3.5. miR-424-5p Targets and Regulates FZD4 to Suppress OS Cell Stemness and Malignant Progression

**Figure 5:**
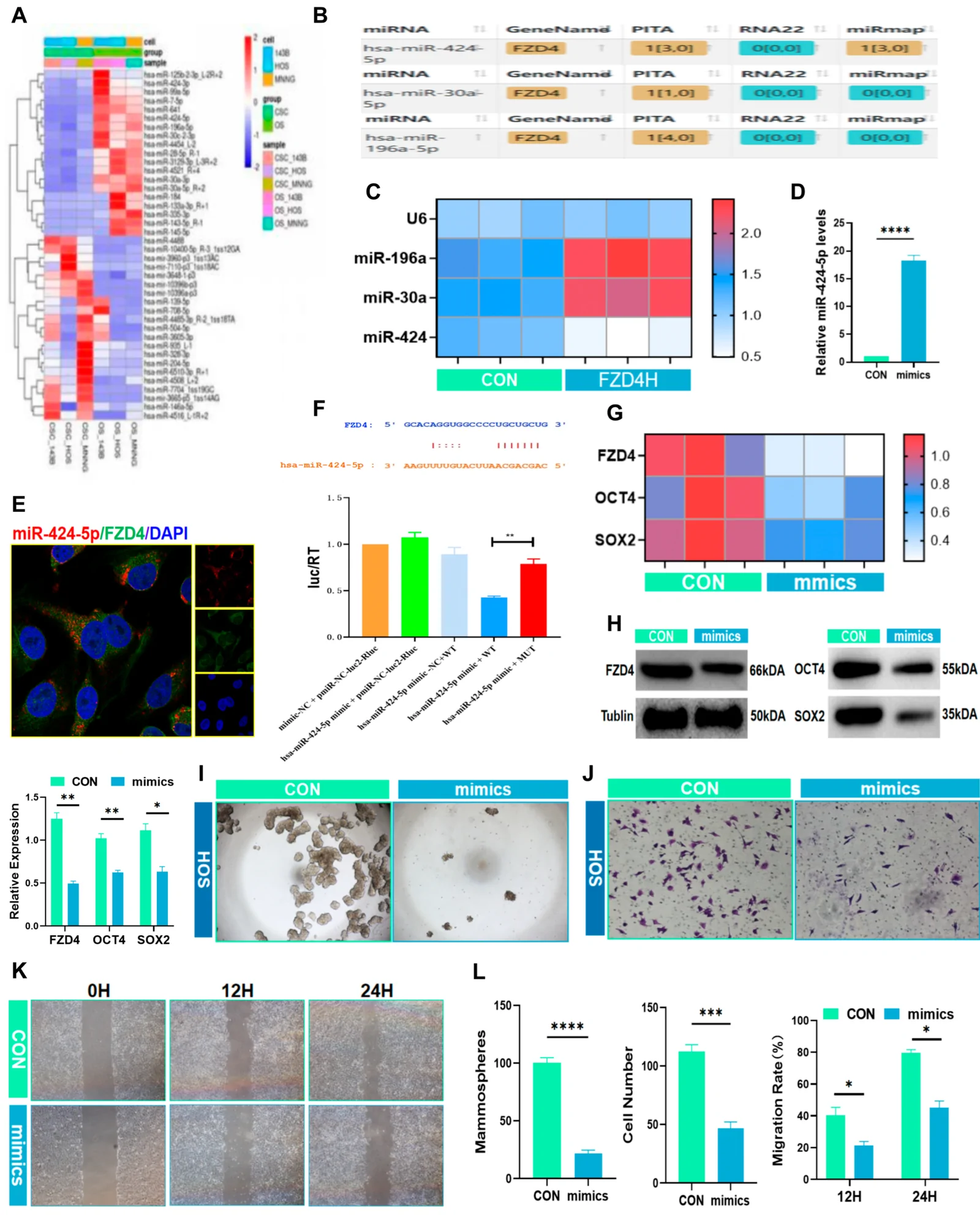
*miR-424-5p* targets and regulates *FZD4* to suppress OS cell stemness and malignant biological behaviors. (**A**) miRNA sequencing heatmap showing differentially expressed miRNAs in OS cells. (**B**) STARBASE analysis showing that *miR-424-5p, miR-30a*, and *miR-196a-5p* may potentially target and bind to *FZD4*. (**C**) qPCR heatmap showing changes in the expression of candidate miRNAs after *FZD4* overexpression. (**D**) qPCR validation of successful mimic transfection. (**E**) FISH assay verifying the colocalization of *miR-424-5p* (red) and FZD4 (green) in OS cells. (**F**) Dual-luciferase reporter assay confirming the direct binding relationship between *miR-424-5p* and the *FZD4* 3′UTR. (**G**) Heatmap showing changes in the mRNA expression levels of *FZD4, OCT4*, and *SOX2* after mimic treatment. (**H**) Western blot analysis of FZD4, OCT4, and SOX2 protein expression levels after mimic treatment (Image S4). (**I**) Sphere-forming ability of OS cells after mimic treatment. (**J**) Transwell assay assessing the effect of *miR-424-5p* mimics on the invasive ability of OS cells. (**K**) Wound-healing assay evaluating the migratory ability of cells in the CON and mimic groups at 0, 12, and 24 h. (**L**) Statistical analysis of the mammosphere number, number of cells penetrating the Transwell membrane, and cell migration rate. Data are presented as means ± SD from three independent experiments (n = 3). * *p* < 0.05, ** *p* < 0.01, *** *p* < 0.001, and **** *p* < 0.0001.

miRNA sequencing analysis showed that multiple differentially expressed miRNAs were aberrantly expressed in OS cells. Since miRNAs exert regulatory effects by binding to mRNAs and suppressing their expression, and *FZD4* was highly expressed in enriched cell spheres, we hypothesized that its interacting miRNAs might be downregulated, thereby reducing their inhibitory effect on FZD4. Combined with prediction using the STARBASE online database, *miR-424-5p*, *miR-30a*, and *miR-196a-5p* were identified as potential upstream regulatory miRNAs of *FZD4* (Figure 5A,B). Further validation showed that *miR-424-5p* expression was significantly decreased in FZD4-overexpressing cell spheres (Figure 5C,D). FISH results demonstrated colocalization of *miR-424-5p* and *FZD4* in cells (Figure 5E and Figure S5). Dual-luciferase reporter assays confirmed that *miR-424-5p* could directly bind to the 3′UTR region of *FZD4* (Figure 5F). qPCR and Western blot analyses showed that treatment with *miR-424-5p* mimics markedly reduced the expression of FZD4, OCT4, and SOX2 (Figure 5G,H and Figure S6). Functional experiments further revealed that *miR-424-5p* mimics significantly inhibited sphere-forming ability, Transwell migration/invasion capacity, and wound-healing ability (Figure 5I–L). Taken together, these results indicate that *miR-424-5p* may suppress OS cell stemness maintenance and malignant phenotypes by targeting and inhibiting *FZD4* expression.

### 3.6. Functional Enrichment and Cell–Cell Communication Analysis of Different OS Cell Subpopulations

**Figure 6:**
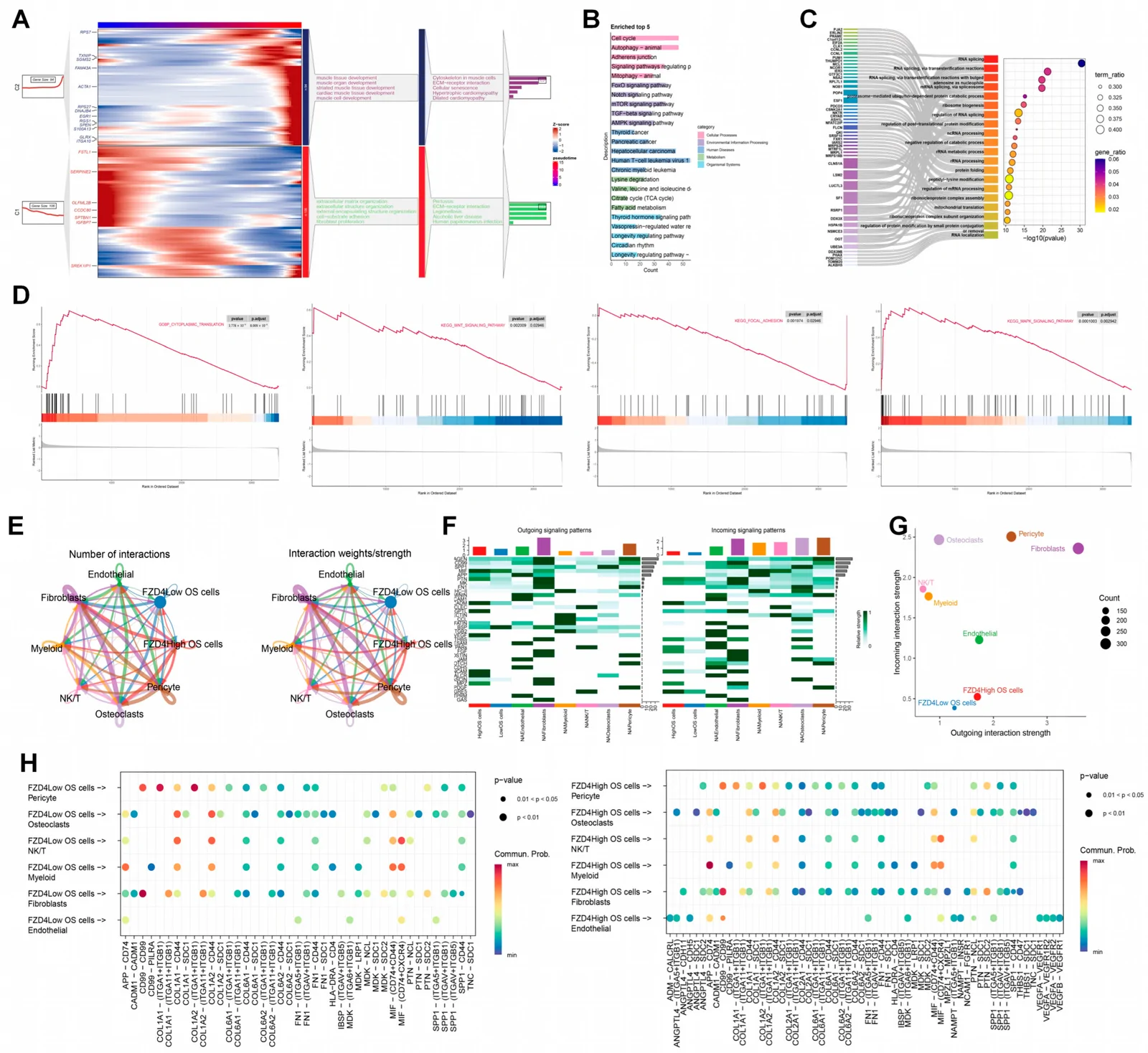
Developmental trajectory, functional enrichment, and cell–cell communication analysis of OS cells. (**A**) Heatmap of dynamically expressed genes associated with pseudotime analysis and branch-dependent gene-clustering analysis, showing expression trends of different gene modules during OS cell development. (**B**) GO enrichment analysis of branch-dependent genes, revealing significant enrichment in extracellular matrix organization, cell migration, angiogenesis, inflammatory response, and stemness-associated biological processes. (**C**) Sankey diagram and KEGG enrichment analysis illustrating the relationship between differentially expressed genes and enriched signaling pathways in OS cells. (**D**) GSEA plots showing representative signaling and biological processes enriched between different OS cell states. (**E**) Cell–cell communication network analysis showing extensive ligand–receptor interactions between OS cells and fibroblasts, endothelial cells, immune cells, and osteoclasts. (**F**) Heatmaps showing outgoing and incoming signaling patterns among different cell populations. (**G**) Scatter plot comparing incoming and outgoing interaction strengths among different cell populations. (**H**) Cell–cell communication bubble plot showing significantly active ligand–receptor signaling pathways among different cell subpopulations.

Pseudotime-dependent gene expression and cell–cell communication analyses were performed to investigate the transcriptional dynamics and tumor microenvironment interactions associated with *FZD4* expression in OS. Branch-dependent heatmap analysis demonstrated dynamic transcriptional remodeling during OS progression, with multiple gene clusters exhibiting distinct expression patterns along developmental trajectories (Figure 6A). GO enrichment analysis revealed that these branch-associated genes were mainly enriched in extracellular matrix organization, cell migration, angiogenesis, inflammatory response, and stemness-associated biological processes (Figure 6B). KEGG enrichment and Sankey analyses revealed that differential genes were closely associated with multiple tumor-promoting pathways, including PI3K–AKT signaling, focal adhesion, cytokine-mediated signaling, and ECM–receptor interaction pathways (Figure 6C). Dynamic trajectory analysis also revealed continuous transcriptional changes in representative genes along pseudotime progression, indicating a substantial progenitor-like state in OS cells (Figure 6D). Further cell–cell communication analysis demonstrated extensive ligand–receptor interactions between OS cells and fibroblasts, endothelial cells, and immune cells (Figure 6E). Among these, MIF, TNF, TGFβ, and CXCL signaling pathways were significantly active in intercellular communication, suggesting their potential involvement in tumor microenvironment remodeling and malignant progression (Figure 6F,G). In addition, the cell–cell communication bubble plot showed that multiple pro-tumorigenic signaling pathways were highly active between OS cells and stromal cells (Figure 6H). Overall, these results suggest that OS contains complex cell–cell communication networks and metabolic reprogramming, which jointly contribute to tumor progression and stemness maintenance.

### 3.7. FZD4 and miR-424-5p Regulate OS Tumorigenesis and Lung Metastasis In Vivo

**Figure 7:**
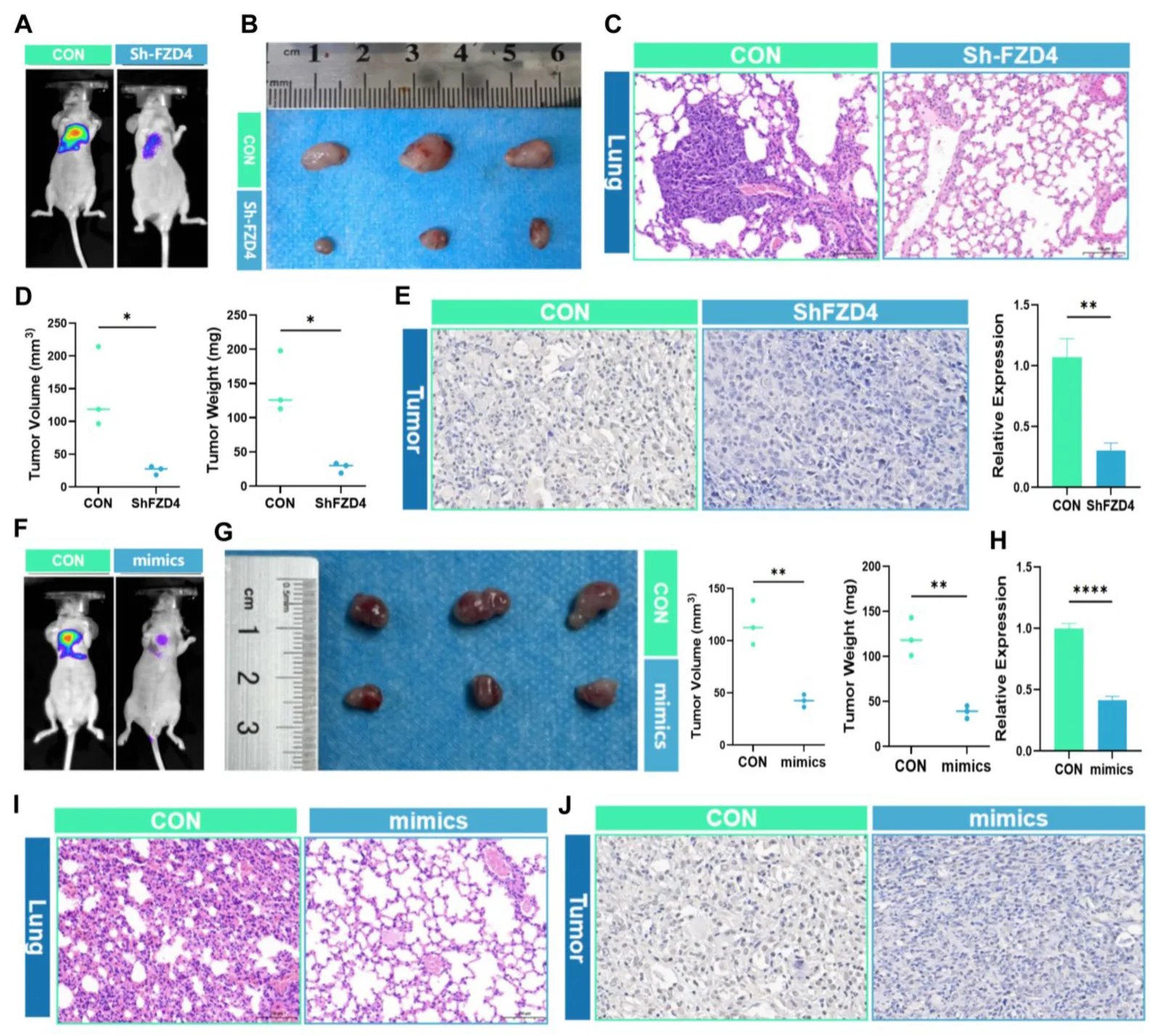
FZD4 and *miR-424-5p* regulate OS tumorigenesis and lung metastasis in vivo. (**A**) In vivo bioluminescence imaging of nude mice showing tumor growth and metastatic signals in the CON and sh-FZD4 groups. (**B**) Representative images of subcutaneous xenograft tumors isolated from mice in the CON and sh-FZD4 groups. (**C**) Hematoxylin and eosin (H&E) staining of lung tissues showing metastatic nodules in the CON and sh-FZD4 groups. (**D**) Statistical analysis of tumor volume and tumor weight in the CON and sh-FZD4 groups. (**E**) Immunohistochemical staining and quantitative analysis of FZD4-related protein expression in tumor tissues from the CON and sh-FZD4 groups. (**F**) In vivo bioluminescence imaging of nude mice showing metastatic signals in the CON and *miR-424-5p* mimic groups. (**G**) Representative images of xenograft tumors and statistical analysis of tumor volume and weight in the CON and miR-424-5p mimic groups. (**H**) Quantitative analysis of relative protein expression levels in tumor tissues following *miR-424-5p* mimic treatment. (**I**) H&E staining of lung tissues showing reduced metastatic lesions in the *miR-424-5p* mimic group compared with the CON group. (**J**) Immunohistochemical staining of tumor tissues from the CON and *miR-424-5p* mimic groups showing decreased positive staining after *miR-424-5p* overexpression. n = 5 mice per group. Data are presented as means ± SD. * *p* < 0.05, ** *p* < 0.01, and **** *p* < 0.0001.

In vivo imaging of nude mice showed that the fluorescence signal was markedly reduced in the sh-FZD4 group compared with the CON group (Figure 7A), indicating that lung metastasis was suppressed. Subcutaneous tumor formation and lung tissue staining showed that both tumor volume and tumor weight were significantly lower in the sh-FZD4 group than in the CON group. Obvious metastatic lesions were observed in the lung tissues of the CON group, whereas lung metastasis was markedly reduced in the sh-FZD4 group (Figure 7B–D and Figure S7). Immunohistochemical analysis further confirmed that the expression of related proteins was significantly decreased in tumor tissues from the sh-FZD4 group (Figure 7E). In addition, after treatment with *miR-424-5p* mimics, the fluorescence signal in nude mice was markedly weakened (Figure 7F), and both tumor volume and tumor weight were significantly reduced (Figure 7G). HE staining showed that lung metastatic lesions were markedly decreased in the mimic group (Figure 7I), accompanied by reduced positive immunohistochemical staining in tumor tissues (Figure 7H,J). Collectively, these results demonstrate that knockdown of FZD4 inhibits OS tumorigenesis and lung metastasis in vivo, while *miR-424-5p* significantly suppresses OS malignant progression by inhibiting *FZD4*.

### 3.8. Restoration of FZD4 Reverses the Inhibitory Effects of miR-424-5p on Stem-like and Malignant Phenotypes of OS Cells

We performed rescue experiments by restoring FZD4 expression in *miR-424-5p* mimic-treated OS cells to further determine whether the inhibitory effects of *miR-424-5p* were functionally mediated through *FZD4*. Successful FZD4 overexpression was confirmed using Western blotting (Supplementary Figure S8). Transwell assays showed that *miR-424-5p* mimics markedly reduced the migratory/invasive capacity of OS cells, whereas restoration of FZD4 substantially reversed this inhibitory effect (Supplementary Figure S9). Similarly, *miR-424-5p* mimics significantly impaired sphere-forming capacity, while FZD4 re-expression partially restored sphere formation (Supplementary Figure S10). Cell proliferation assays further demonstrated reduced proliferative activity following *miR-424-5p* overexpression, which was rescued by FZD4 restoration (Supplementary Figure S11). Consistently, flow cytometric analysis showed that the reduction in the stemness-associated cell population induced by *miR-424-5p* was reversed following FZD4 re-expression (Supplementary Figure S12). At the molecular level, Western blot analysis demonstrated that *miR-424-5p* mimics decreased FZD4 and β-catenin expression together with the stemness-associated transcription factors OCT4 and SOX2. Restoration of FZD4 substantially recovered β-catenin, OCT4, and SOX2 expression (Supplementary Figure S13). Immunofluorescence analysis further supported the corresponding restoration of β-catenin signaling following FZD4 re-expression (Supplementary Figure S14).

Collectively, these rescue experiments provide functional evidence that FZD4 is an important mediator of the inhibitory effects of *miR-424-5p* on stem-like and malignant phenotypes of OS cells and support the involvement of FZD4-associated Wnt/β-catenin signaling.

## 4. Discussion

OS is a highly aggressive primary malignant bone tumor characterized by marked tumor heterogeneity, early metastasis, therapeutic resistance, and poor prognosis [2,3]. Although comprehensive treatment strategies, including surgery, chemotherapy, targeted therapy, and immunotherapy, have improved the survival of some patients, the prognosis of patients with recurrent or metastatic OS remains unsatisfactory [4,5,29]. Therefore, elucidating novel molecular mechanisms that drive OS progression and maintenance of stem-like properties is essential for developing more effective therapeutic strategies.

Tumor heterogeneity and cancer stemness are key biological features driving OS progression, metastasis, recurrence, and therapeutic resistance [6,7]. In this study, single-cell transcriptomic analysis revealed pronounced cellular heterogeneity within the OS tumor microenvironment, identifying multiple cell populations, including OS cells, tumor-associated macrophages, fibroblasts, osteoclasts, natural killer/T cells, endothelial cells, and pericytes [28,29]. Further reclustering of OS cells identified tumor cell subpopulations with distinct developmental potentials. Among these, some subpopulations exhibited higher developmental potential and a less differentiated state, suggesting the presence of stem-like tumor cell populations in OS. These results indicate that OS progression may be driven by tumor cell subpopulations with enhanced developmental potential and stemness-associated characteristics.

FZD4 is a member of the frizzled receptor family and an important receptor in the Wnt signaling pathway [30]. Wnt signaling is widely involved in embryonic development, tissue homeostasis, stem cell maintenance, and tumor progression [31]. In this study, FZD4 was enriched in OS cell populations with high developmental potential and positively correlated with stemness scores. Pseudotime trajectory analysis further showed that FZD4-high cells were mainly distributed in early developmental states, whereas FZD4-low cells were predominantly located in terminally differentiated regions. These results suggest that FZD4 may participate in maintaining the undifferentiated state and developmental plasticity of OS cells, which is consistent with previous evidence linking FZD4/Wnt signaling to cancer stemness and invasiveness.

Clinical validation further supported the biological and clinical significance of FZD4 in OS. Immunohistochemical staining showed that the expression levels of FZD4, OCT4, and SOX2 were higher in OS tissues than in normal bone tissues; qPCR and Western blot analyses also confirmed the upregulation of these molecules in tumor tissues. In addition, patients with high FZD4 expression had shorter overall survival, suggesting that FZD4 may serve as a potential prognostic indicator for OS. As classical stemness-associated transcription factors, OCT4 and SOX2 were coordinately upregulated with FZD4 [31,32], further indicating that FZD4 is closely associated with the activation of stemness-related programs in OS.

Functional experiments confirmed that FZD4 plays an important role in promoting malignant phenotypes in OS cells [21]. FZD4 was highly expressed in OS cell lines, and enriched tumor spheres exhibited further increases in FZD4, OCT4, and SOX2 expression, accompanied by enhanced sphere-forming and invasive abilities [33]. Conversely, FZD4 knockdown significantly downregulated OCT4 and SOX2 expression, inhibited sphere-forming ability, and weakened cell migration and invasion. These findings indicate that FZD4 is a marker of stem-like OS cells as well as a functional regulator that maintains stemness and promotes migration and invasion. Therefore, FZD4 may represent a promising therapeutic target for suppressing OS stemness and metastatic potential [25].

MicroRNAs are important post-transcriptional regulators that participate in tumor progression by binding to target mRNAs and suppressing their expression [34]. In this study, miRNA sequencing and STARBASE prediction were used to screen and validate candidate miRNAs, revealing that *miR-424-5p* was significantly downregulated in FZD4-overexpressing tumor spheres. FISH assays confirmed the colocalization of *miR-424-5p* and FZD4 in OS cells. Dual-luciferase reporter assays demonstrated that *miR-424-5p* directly binds to the *FZD4* 3′UTR, indicating that *miR-424-5p* directly targets and regulates *FZD4*. Functionally, *miR-424-5p* mimics significantly reduced the expression of FZD4, OCT4, and SOX2, and inhibited sphere-forming ability, Transwell invasion, and wound-healing capacity. These results suggest that downregulation of *miR-424-5p* may relieve its inhibitory effect on FZD4, thereby promoting FZD4-mediated stemness maintenance and malignant progression. Thus, the *miR-424-5p/FZD4* axis may represent an important regulatory mechanism underlying OS stemness and invasiveness.

A major finding of this study is the establishment of a functional link between miR-424-5p and FZD4. Although direct binding of *miR-424-5p* to the *FZD4* 3′UTR was demonstrated with the dual-luciferase reporter assay, direct binding alone does not establish that *FZD4* mediates the biological effects of *miR-424-5p*. To address this issue, we performed rescue experiments in which FZD4 expression was restored in *miR-424-5p* mimic-treated OS cells. FZD4 restoration substantially reversed the inhibitory effects of miR-424-5p on sphere formation, migration/invasion, proliferation, and stemness-associated cell populations. These findings establish FZD4 as an important functional downstream mediator of *miR-424-5p* in OS cells.

Our additional experiments provide further evidence linking this regulatory axis to Wnt/β-catenin signaling. FZD4 depletion reduced β-catenin and stemness-associated proteins, whereas miR-424-5p overexpression similarly decreased FZD4, β-catenin, OCT4, and SOX2. Restoration of FZD4 rescued β-catenin and stemness-associated protein expression. These findings support a model in which *miR-424-5p* suppresses FZD4-dependent Wnt/β-catenin signaling, thereby attenuating stem-like and malignant phenotypes in OS cells. Nevertheless, the Wnt signaling network is complex, and additional studies using pathway-specific inhibitors, TCF/LEF transcriptional reporters, and comprehensive downstream target analyses will be required to fully define the canonical and non-canonical components involved.

The tumor microenvironment is a key determinant of OS progression and therapeutic response [35]. Previous studies have emphasized that interactions between tumor cells and stromal or immune components can promote tumor growth, metastasis, immune escape, and therapeutic resistance [36]. In this study, GO and KEGG enrichment analyses showed that differentially expressed genes were significantly enriched in pathways related to cell migration, cell adhesion, extracellular matrix remodeling, inflammatory response, and angiogenesis. Cell–cell communication analysis further revealed extensive ligand–receptor interactions between OS cells and fibroblasts, endothelial cells, immune cells, and osteoclasts [37,38]. Cell–cell communication analysis predicted extensive ligand–receptor interactions between OS cells and fibroblasts, endothelial cells, immune cells, and osteoclasts. These computational findings suggest that FZD4-associated OS cell states may be linked to distinct patterns of tumor–microenvironment communication. However, because CellChat analysis is inferential and no direct functional manipulation of stromal or immune cells was performed, these findings should be interpreted as hypothesis-generating rather than as direct evidence that the miR-424-5p/FZD4 axis remodels the tumor microenvironment [39]. In addition, metabolic pathway analysis showed differences among different cell subpopulations in amino acid metabolism, lipid metabolism, glycolysis, and other metabolic processes, indicating that OS progression is accompanied by altered intercellular communication as well as metabolic reprogramming [40]. Given that stem-like tumor cells often possess strong metabolic plasticity, FZD4-associated tumor cell populations may jointly promote OS progression by coordinately regulating stemness programs, microenvironmental communication, and metabolic adaptation [36,41].

In vivo experiments further confirmed the pathological significance of FZD4 and *miR-424-5p* in OS progression. FZD4 knockdown significantly inhibited tumor growth in nude mice, reduced tumor weight, weakened bioluminescent signals, and decreased lung metastatic lesions. Immunohistochemical staining showed decreased expression of related proteins in tumor tissues from the sh-FZD4 group. Similarly, treatment with *miR-424-5p* mimics markedly inhibited tumor growth and lung metastasis in vivo and reduced immunohistochemically positive signals in tumor tissues. These results demonstrate that FZD4 promotes OS tumorigenesis and lung metastasis in vivo, whereas *miR-424-5p* suppresses OS malignant progression, at least partly, by targeting FZD4.

This study has several limitations. First, although the findings were validated using more than one OS cell model, additional validation in a broader panel of molecularly heterogeneous OS cell lines and patient-derived models would further strengthen their generalizability. Second, although our rescue experiments support a functional miR-424-5p/FZD4/β-catenin regulatory axis, the precise downstream Wnt signaling components remain incompletely defined. Additional experiments involving β-catenin-specific inhibition, TCF/LEF transcriptional activity, and downstream Wnt target genes will be required to further delineate this mechanism. Third, the stemness-related assays used in this study, including sphere formation, stemness-associated transcription factors, flow cytometry, and computational developmental potential analyses, primarily support a stem-like phenotype. Serial sphere formation, limiting dilution, and tumor initiation assays will be required to definitively establish cancer stem cell properties [19,20,21,24,25,31]. Fourth, CellChat analysis provides computational predictions of intercellular communication rather than direct functional evidence of tumor microenvironment remodeling; therefore, future co-culture and in vivo microenvironment studies are warranted. Fifth, larger independent clinical cohorts with complete clinicopathological information are required to determine whether FZD4 has independent prognostic value. Finally, the safety, stability, delivery efficiency, and clinical feasibility of miR-424-5p-based therapeutic strategies require further investigation [16,34,35,36].

In summary, this study systematically demonstrates that FZD4 is a stemness-associated oncogenic regulator in OS. FZD4 is enriched in OS cell subpopulations with high developmental potential and promotes stemness maintenance, migration, invasion, tumorigenesis, and lung metastasis. Mechanistically, *miR-424-5p* directly targets *FZD4* and suppresses FZD4-mediated malignant phenotypes. These findings provide new insights into the molecular mechanisms underlying OS stemness regulation and tumor microenvironment remodeling and suggest that the miR-424-5p/FZD4 axis may serve as a potential biomarker and therapeutic target for OS.

## 5. Conclusions

This study identifies FZD4 as an important regulator associated with developmental potential and stem-like phenotypes in OS cells. *miR-424-5p* directly targets the *FZD4* 3′UTR and suppresses FZD4 expression. Rescue experiments demonstrate that restoration of FZD4 substantially reverses the inhibitory effects of *miR-424-5p* on sphere formation, proliferation, migration/invasion, and stemness-associated phenotypes. These effects are accompanied by corresponding changes in β-catenin, OCT4, and SOX2, supporting the involvement of FZD4-associated Wnt/β-catenin signaling. Collectively, these findings establish *FZD4* as an important functional mediator of *miR-424-5p* activity in OS cells and provide a stronger mechanistic basis for further investigation into the miR-424-5p/FZD4 axis as a potential therapeutic target.

## Data Availability

The transcriptomic sequencing data and batch data of human OS in this study were obtained from the GEO database (GSE152048). The original miRNA sequencing data of OS cells in this study can be provided by the authors upon request.

## Supplementary Materials

The following supporting information can be downloaded at https://www.mdpi.com/article/10.3390/cancers18172846/s1. Supplementary Figure S1. Flow cytometry analysis of the CD133^+^/CD44^+^ stemness-associated cell populations in CON and FZD4-overexpressing (FZD4H) cells. Representative flow cytometric plots showing the proportion of stem-like cell populations after FZD4 overexpression. Supplementary Figure S2. Representative mammosphere formation images of 143B cells in the CON and FZD4H groups. FZD4 overexpression significantly enhanced sphere formation ability and stem-like characteristics of osteosarcoma cells. Supplementary Figure S3. qPCR analysis of stemness-related genes in 143B cells following FZD4 overexpression. Relative expression levels of FZD4, NANOG, OCT4, and SOX2 were significantly increased in the FZD4H group compared with the CON group. Supplementary Figure S4. Western blot analysis of β-catenin and stemness-associated proteins in CON and Sh-FZD4 osteosarcoma cells. Knockdown of FZD4 decreased β-catenin, OCT4, and NANOG expression levels (Image S5). Supplementary Figure S5. FISH analysis showing the co-localization of miR-424-5p (red) and FZD4 (green) in 143B cells. DAPI (blue) was used for nuclear staining. Supplementary Figure S6. qPCR analysis of stemness-associated genes in 143B cells following miR-424-5p mimics treatment. Relative expression levels of FZD4, NANOG, OCT4, and SOX2 were significantly decreased in the mimics group compared with the CON group. Supplementary Figure S7. Representative xenograft tumors and quantitative analysis of tumor volume and tumor weight in nude mice injected with CON or FZD4-knockdown 143B cells. FZD4 depletion significantly inhibited in vivo tumor growth. Supplementary Figure S8. Validation of FZD4 overexpression efficiency in osteosarcoma cells. (A) qPCR analysis of FZD4 mRNA expression in control (CON) and FZD4-overexpressing (Oe-FZD4) osteosarcoma cells. (B) Representative Western blot images and quantitative analysis of FZD4 protein expression in CON and Oe-FZD4 cells (Image S6). Data are presented as the mean ± SD. *** *p* < 0.001, **** *p* < 0.0001. Supplementary Figure S9. FZD4 restoration rescues the inhibitory effect of miR-424-5p on the migration/invasion ability of osteosarcoma cells. Representative Transwell images and quantitative analysis of migrated/invaded cells in the control (CON), miR-424-5p mimic (mimics), and miR-424-5p mimic plus FZD4 overexpression (mi+Oe-FZD4) groups. Data are presented as the mean ± SD. *** *p* < 0.001, **** *p* < 0.0001. Supplementary Figure S10. FZD4 restoration rescues the inhibitory effect of miR-424-5p on the sphere-forming capacity of osteosarcoma cells. Representative images and quantitative analysis of sphere formation in the control (CON), miR-424-5p mimic (mimics), and miR-424-5p mimic plus FZD4 overexpression (mi+Oe-FZD4) groups. Data are presented as the mean ± SD. ** *p* < 0.01, *** *p* < 0.001. Supplementary Figure S11. FZD4 restoration rescues the inhibitory effect of miR-424-5p on osteosarcoma cell proliferation. Cell proliferation was assessed using the CCK-8 assay in the control (CON), miR-424-5p mimic (mimics), and miR-424-5p mimic plus FZD4 overexpression (mi+Oe-FZD4) groups at 0, 24, 48, and 72 h. Cell viability was determined by measuring absorbance at 450 nm. Data are presented as the mean ± SD. Supplementary Figure S12. FZD4 restoration rescues the inhibitory effect of miR-424-5p on the CD133^+^/CD44^+^ stemness-associated cell populations in osteosarcoma cells. Representative flow cytometry plots showing the distribution of stemness-associated cell populations in the control (CON), miR-424-5p mimic (mimics), and miR-424-5p mimic plus FZD4 overexpression (mi+Oe-FZD4) groups. Supplementary Figure S13. FZD4 restoration reverses miR-424-5p-mediated suppression of β-catenin and stemness-associated proteins in osteosarcoma cells. (A) Representative Western blot images showing the protein expression levels of β-catenin, FZD4, OCT4, and SOX2 in the control (CON), miR-424-5p mimic (mimics), and miR-424-5p mimic plus FZD4 overexpression (mimics+Oe-FZD4) groups (Image S7). (B) Quantitative analysis of the relative protein expression levels normalized to Tubulin. Data are presented as the mean ± SD. * *p* < 0.05, ** *p* < 0.01, *** *p* < 0.001. Supplementary Figure S14. FZD4 restoration reverses miR-424-5p-mediated suppression of β-catenin in osteosarcoma cells. Representative immunofluorescence images showing β-catenin expression in the control (CON), miR-424-5p mimic (mimics), and miR-424-5p mimic plus FZD4 overexpression (mi+Oe-FZD4) groups. β-catenin is shown in green, and nuclei were counterstained with DAPI (blue). Supplementary Figure S15. Flow cytometry gating strategy. The main cell population was identified based on forward scatter (FSC-H) and side scatter (SSC-H) characteristics. The E1 gate was established to exclude cellular debris and non-target events, and approximately 62.92% of total events were included within the selected population for subsequent flow cytometric analysis. Supplementary File S1: Original uncropped Western blot images corresponding to Figure 3E, Figure 4D,I and Figure 5H and Supplementary Figures S4, S8 and S13.
