# Supplementary material for: miR-424-5p Regulates Stem-like and Malignant Phenotypes in Osteosarcoma by Targeting FZD4
Source: Cancers (Basel). 2026 Sep 3;18(17):2846. doi: 10.3390/cancers18172846 (PMC13564835; doi:10.3390/cancers18172846)
Supplement: Supplementary file 1 [file cancers-18-02846-s001.zip › cancers-4426132-File S1.pdf]

Image S1: Figure 3.E

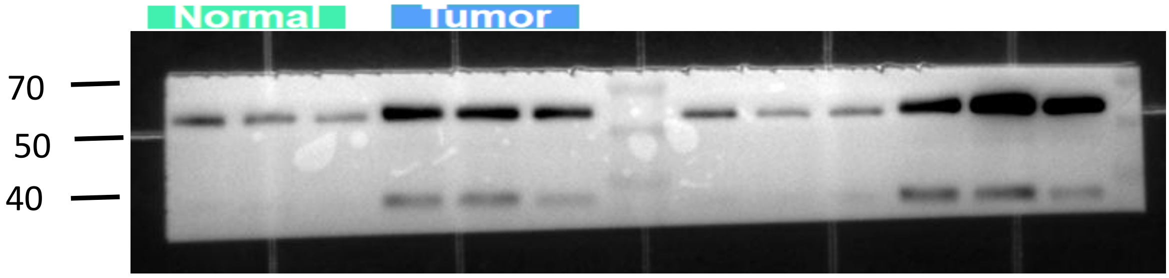

60.468、51.379、46.627、136.923、131.121、109.208

kD

FZD4(60，66kDa）

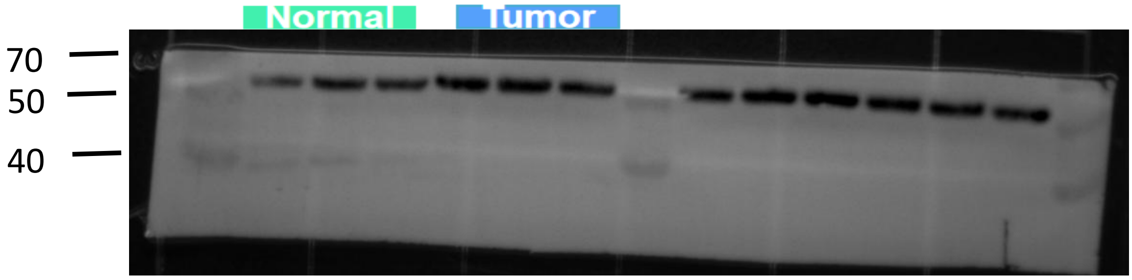

kD

17.775、24.458、22.601、46.186、45.161、35.508

OCT4(55kDa）

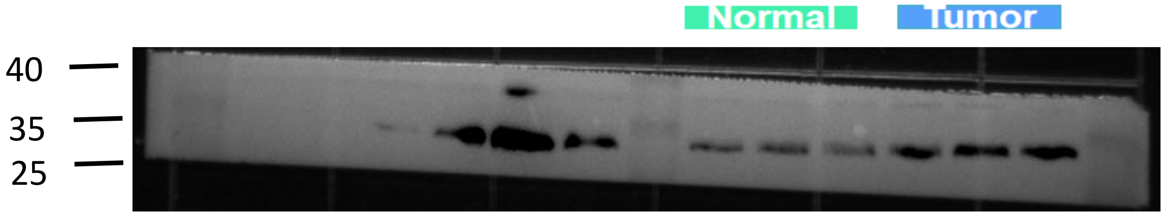

21.181、24.689、20.602、40.120、45.323、48.957

kD

SOX2(34-40kDa）

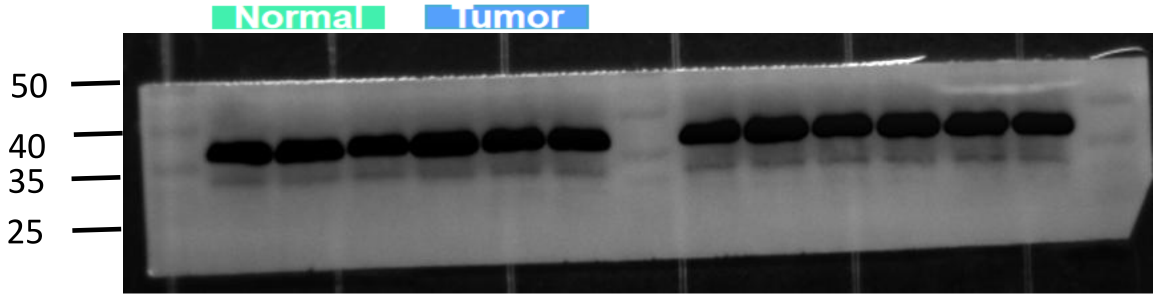

43.108、57.468、51.561、59.304、48.231、55.822

kD

GAPDH(36kDa）

Image S2: Figure 4.D

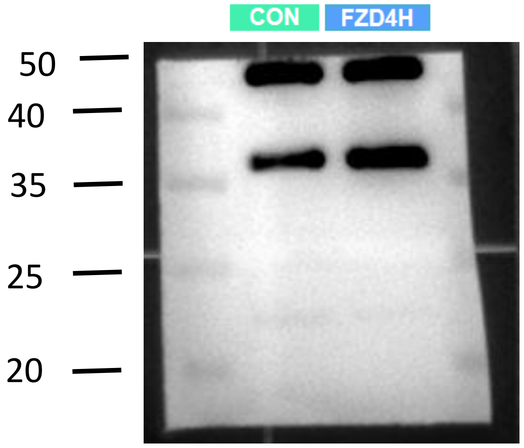

kD

119.295、126.874、

75.660、127.789

Tublin(50kDa），SOX2（34-40kDa）

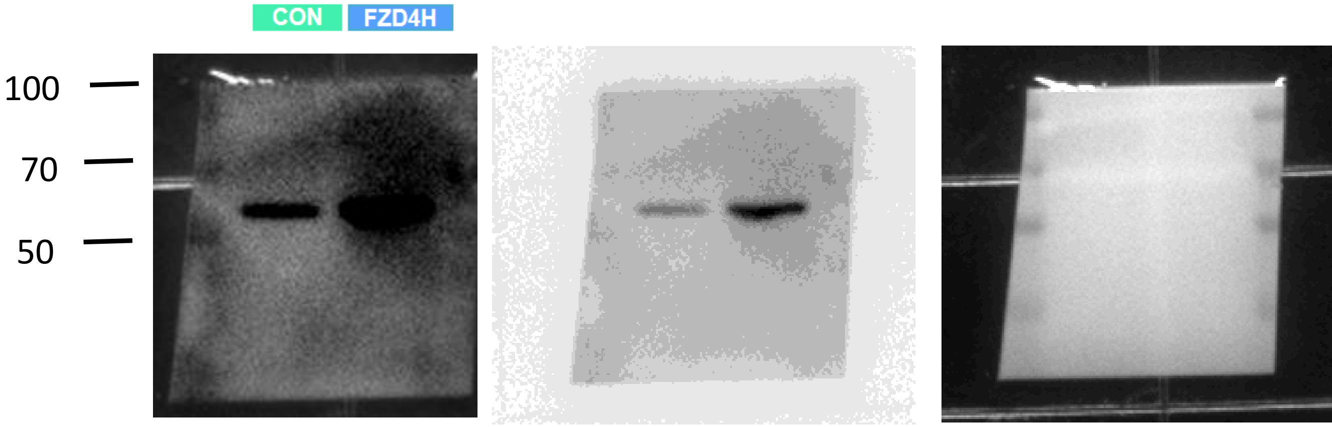

52.193、112.442、

kD

OCT4（55kDa）

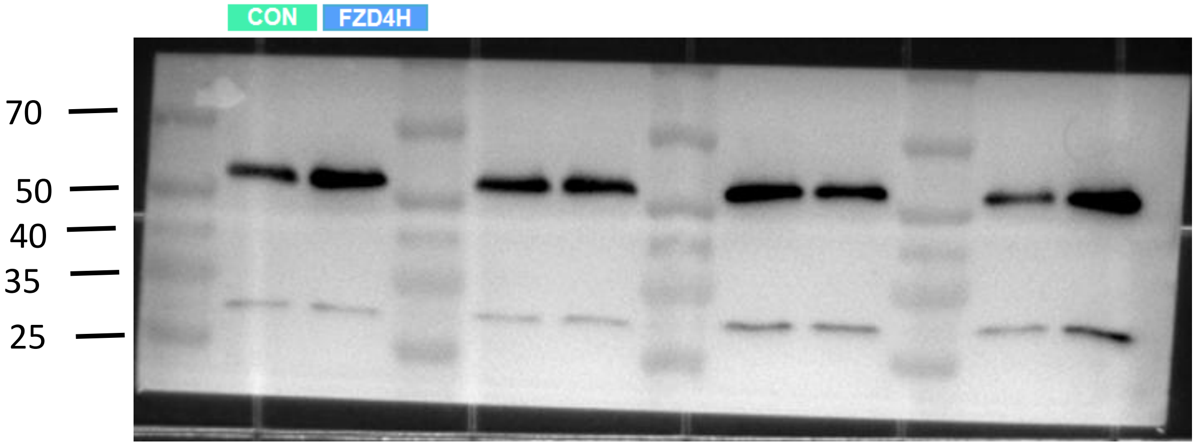

kD

44.506、87.590

FZD4（60kDa，66kDa）

Image S3: Figure 4.I

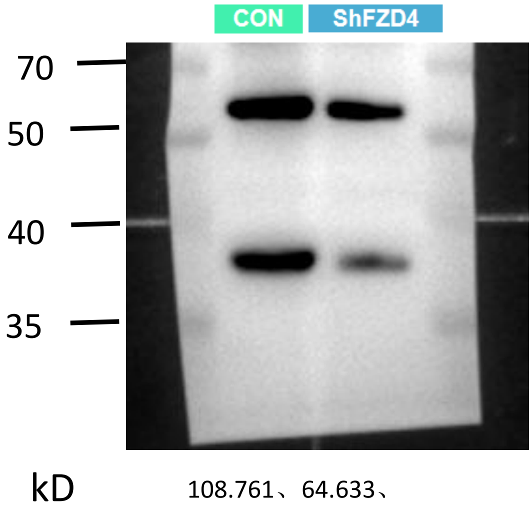

108.761、64.633、
89.465、34.158

OCT4（55kDa），SOX2（34-40kDa）

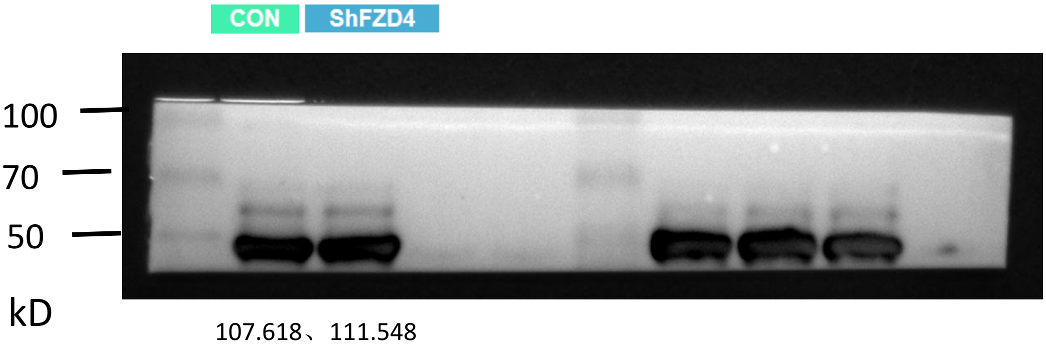

107.618、111.548

Tublin(50kDa）

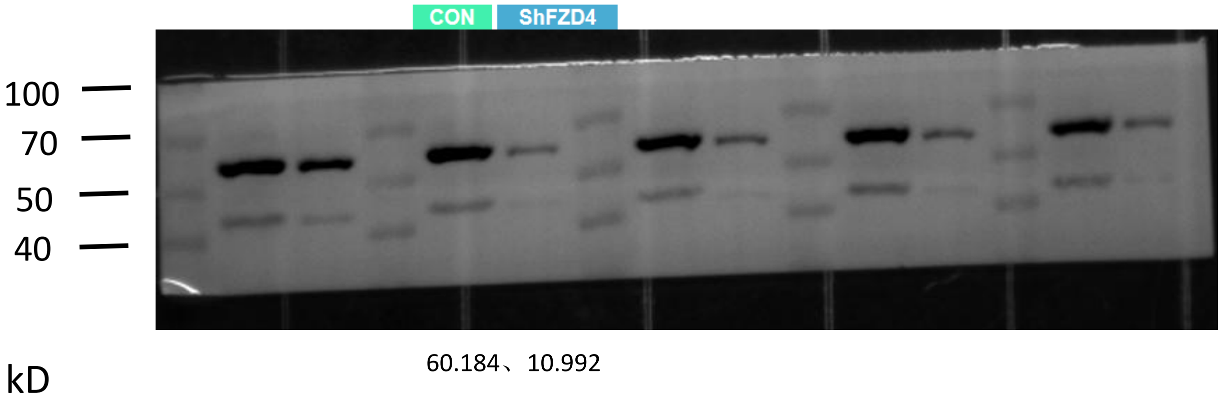

60.184、10.992

FZD4（60kDa，66kDa）

Image S4: Figure 5.H

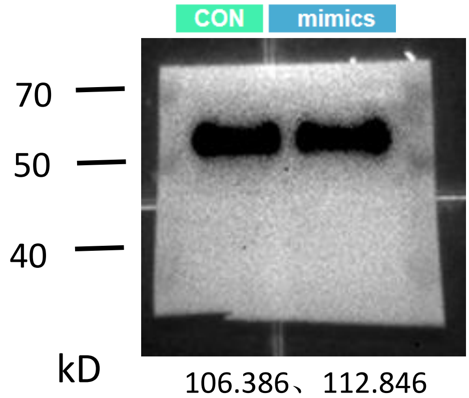

106.386、112.846

Tublin(50kDa）

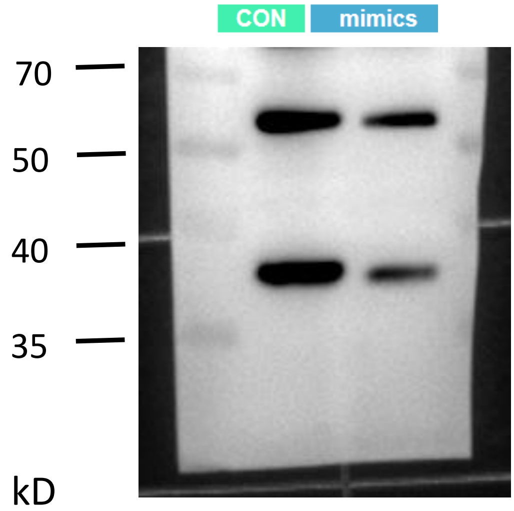

96.354、62.188

131.174、50.748

OCT4（55kDa），SOX2（34-40kDa）

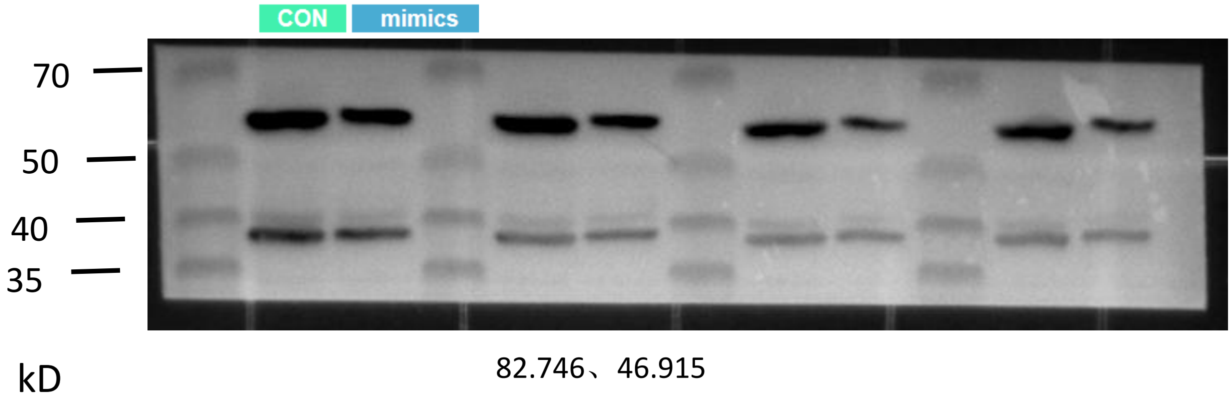

82.746、46.915

FZD4（60kDa，66kDa）

Image S5: Supplementary Figure S4

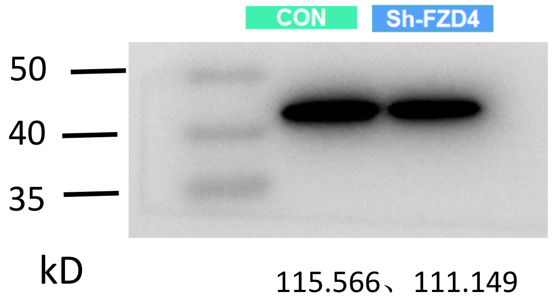

115.566、111.149

β -actin（42kDa）

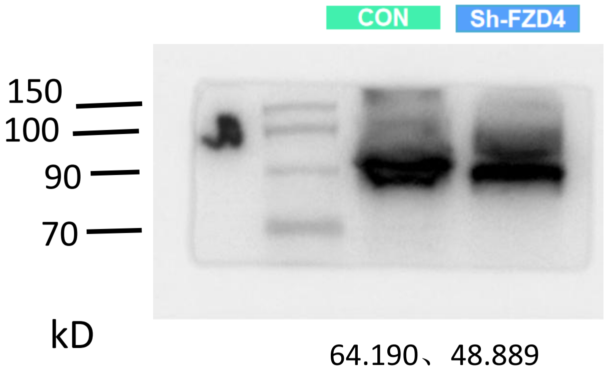

64.190、48.889

β -catenin（92kDa）

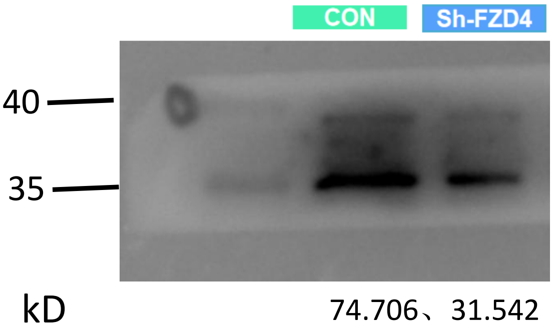

74.706、31.542

NANOG（34kDa）

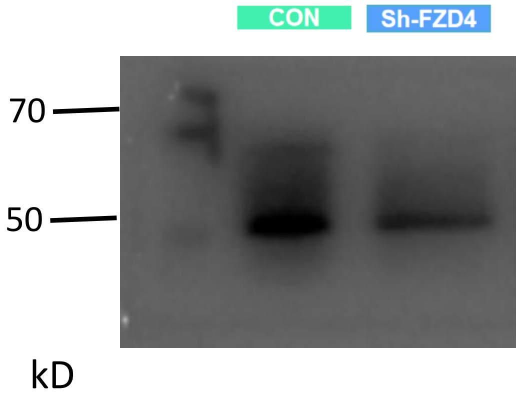

64.037、33.752

OCT4（55kDa）

Image S6: Supplementary Figure S8

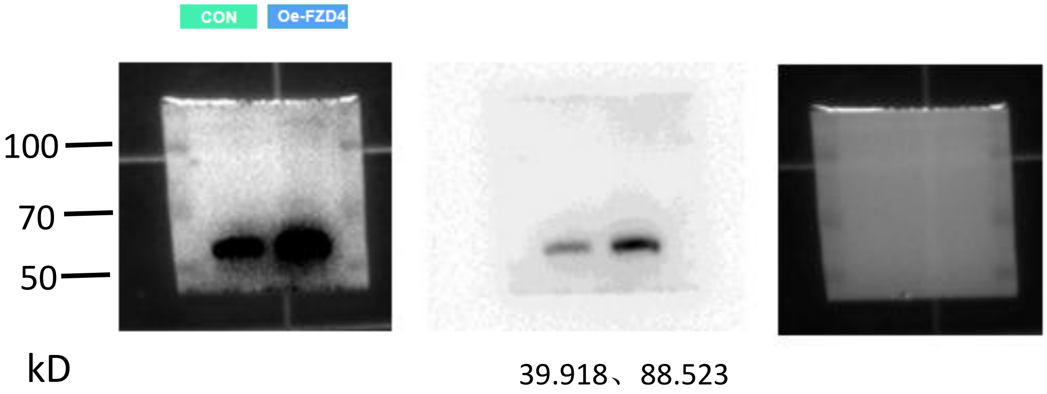

FZD4（60kDa，66kDa）

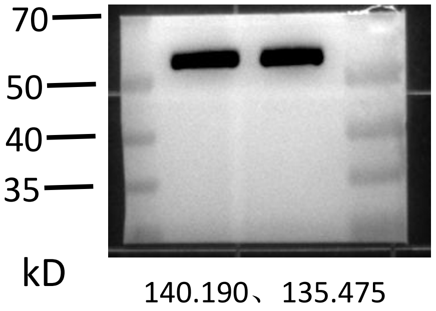

Tublin（50kDa）

Image S7: Supplementary Figure S13

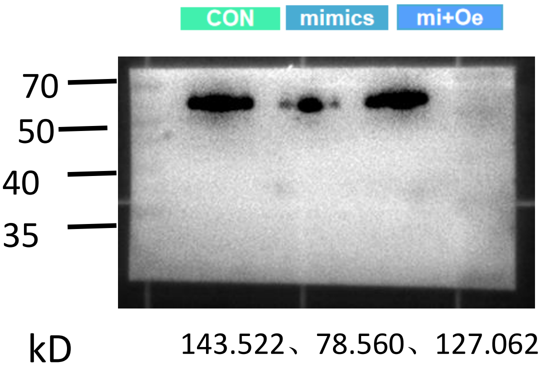

FZD4（60kDa，66kDa）

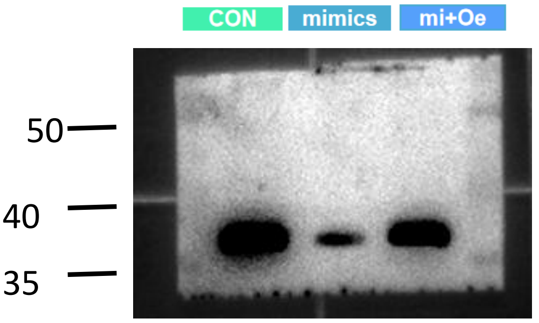

kD 159.998、51.763、123.237

SOX2（34-40kDa）

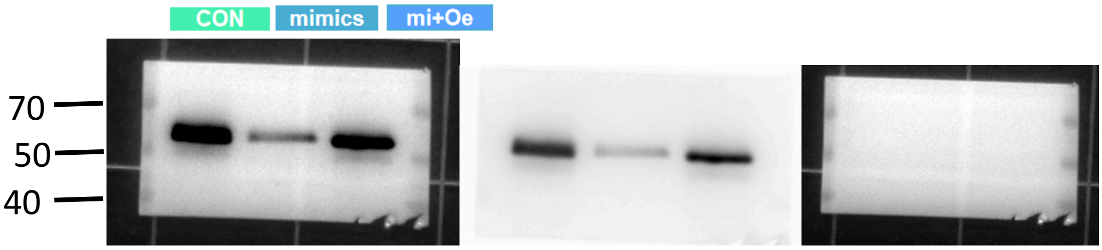

kD

151.840、62.677、130.354

OCT4（55kDa）

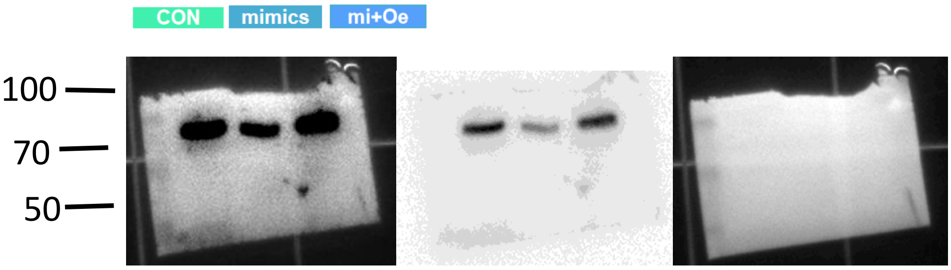

kD

74.741、45.166、73.952

β -catenin（92kDa）

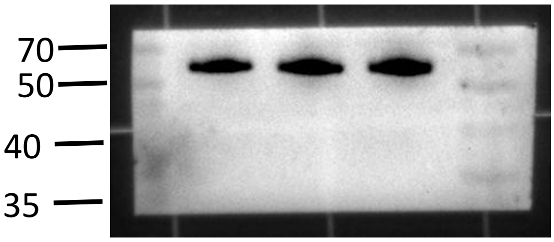

123.916、138.516、139.205

kD

Tublin（50kDa）
