# Supplementary material for: miR-424-5p Regulates Stem-like and Malignant Phenotypes in Osteosarcoma by Targeting FZD4
Source: Cancers (Basel). 2026 Sep 3;18(17):2846. doi: 10.3390/cancers18172846 (PMC13564835; doi:10.3390/cancers18172846)
Supplement: Supplementary file 1 [file cancers-18-02846-s001.zip › cancers-4426132-supplementary figures.pdf]

Supplementary Materials

Supplementary Figure S1

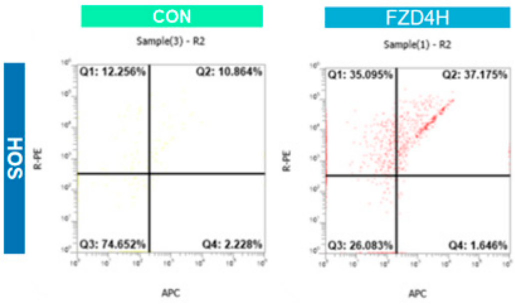

Supplementary Figure S1. Flow cytometry analysis of the CD133⁺/CD44⁺ stemness-associated cell populations in CON and FZD4-overexpressing (FZD4H) cells. Representative flow cytometric plots showing the proportion of stem-like cell populations after FZD4 overexpression.

Supplementary Figure S2

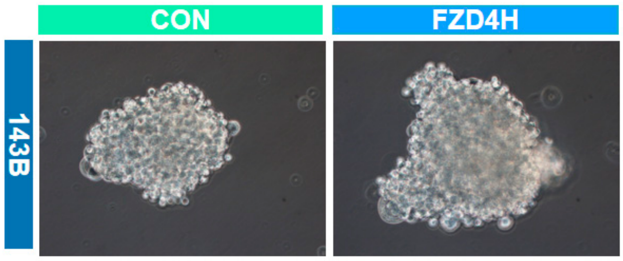

Supplementary Figure S2. Representative mammosphere formation images of 143B cells in the CON and FZD4H groups. FZD4 overexpression significantly enhanced sphere formation ability and stem-like characteristics of osteosarcoma cells.

Supplementary Figure S3

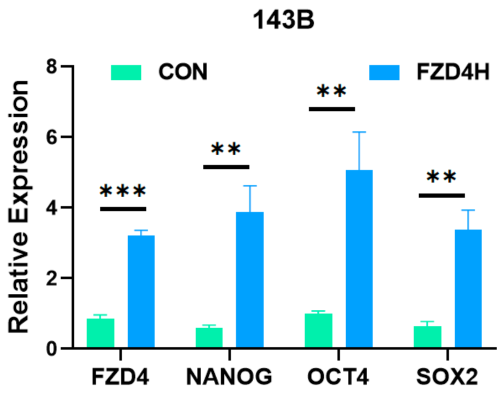

Supplementary Figure S3. qPCR analysis of stemness-related genes in 143B cells following FZD4 overexpression. Relative expression levels of FZD4, NANOG, OCT4, and SOX2 were significantly increased in the FZD4H group compared with the CON group.

Supplementary Figure S4

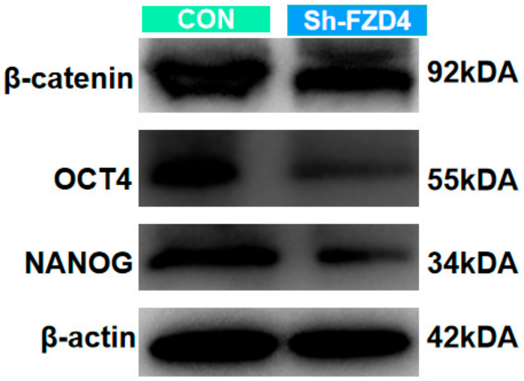

Supplementary Figure S4. Western blot analysis of β-catenin and stemness-associated proteins in CON and Sh-FZD4 osteosarcoma cells. Knockdown of FZD4 decreased β-catenin, OCT4, and NANOG expression levels (Image S5).

Supplementary Figure S5

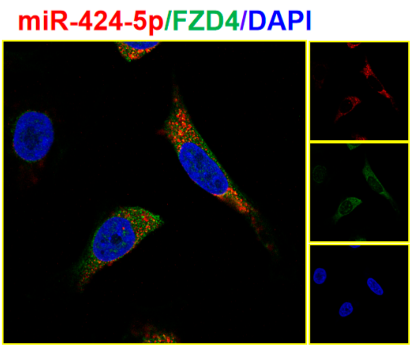

Supplementary Figure S5. FISH analysis showing the co-localization of miR-424-5p (red) and FZD4 (green) in 143B cells. DAPI (blue) was used for nuclear staining.

Supplementary Figure S6

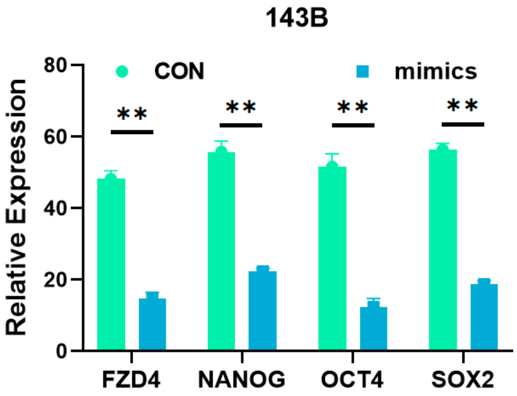

Supplementary Figure S6. qPCR analysis of stemness-associated genes in 143B cells following miR-424-5p mimics treatment. Relative expression levels of FZD4, NANOG, OCT4, and SOX2 were significantly decreased in the mimics group compared with the CON group.

Supplementary Figure S7

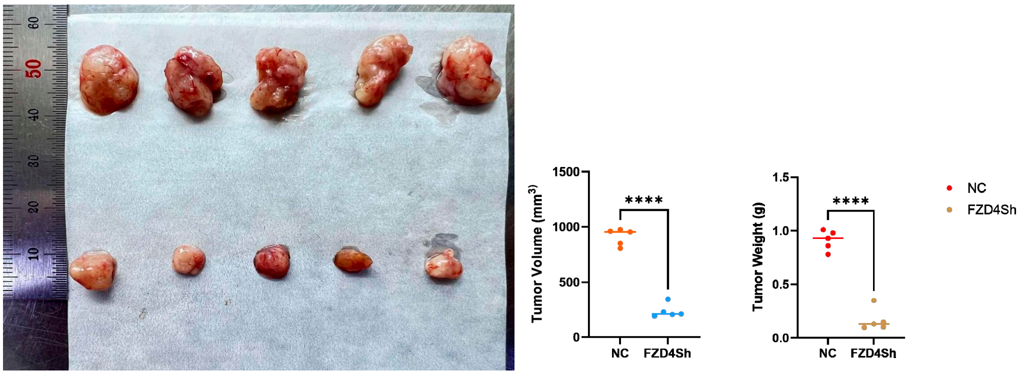

Supplementary Figure S7. Representative xenograft tumors and quantitative analysis of tumor volume and tumor weight in nude mice injected with CON or FZD4-knockdown 143B cells. FZD4 depletion significantly inhibited in vivo tumor growth.

Supplementary Figure S8

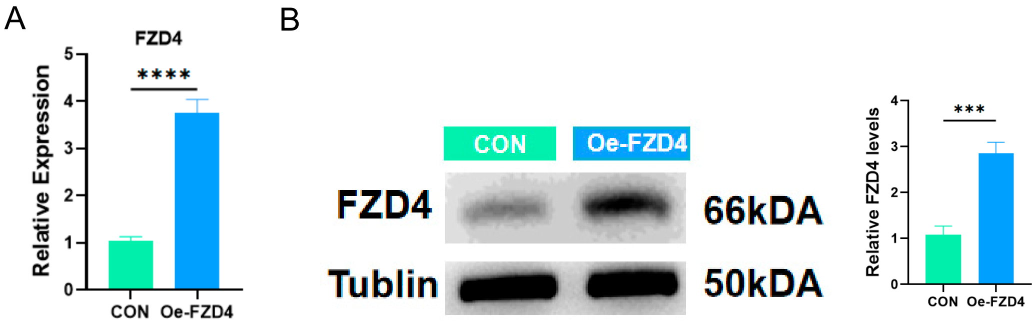

Supplementary Figure S8. Validation of FZD4 overexpression efficiency in osteosarcoma cells. (A) qPCR analysis of FZD4 mRNA expression in control (CON) and FZD4-overexpressing (Oe-FZD4) osteosarcoma cells. (B) Representative Western blot images and quantitative analysis of FZD4 protein expression in CON and Oe-FZD4 cells (Image S6). Data are presented as the mean ± SD. ***$p < 0.001$, ****$p < 0.0001$.

Supplementary Figure S9

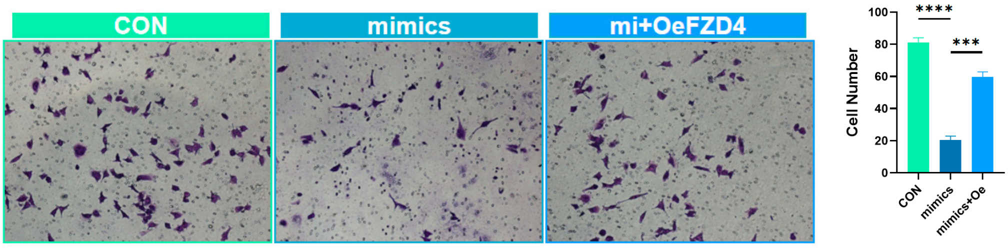

Supplementary Figure S9. FZD4 restoration rescues the inhibitory effect of miR-424-5p on the migration/invasion ability of osteosarcoma cells. Representative Transwell images and quantitative analysis of migrated/invaded cells in the control (CON), miR-424-5p mimic (mimics), and miR-424-5p mimic plus FZD4 overexpression (mi+Oe-FZD4) groups. Data are presented as the mean ± SD. ***$p < 0.001$, ****$p < 0.0001$

Supplementary Figure S10

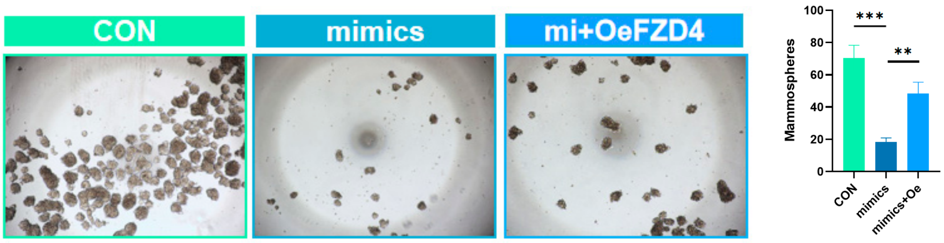

Supplementary Figure S10. FZD4 restoration rescues the inhibitory effect of miR-424-5p on the sphere-forming capacity of osteosarcoma cells. Representative images and quantitative analysis of sphere formation in the control (CON), miR-424-5p mimic (mimics), and miR-424-5p mimic plus FZD4 overexpression (mi+Oe-FZD4) groups. Data are presented as the mean ± SD. **$p < 0.01$, ***$p < 0.001$.

Supplementary Figure S11

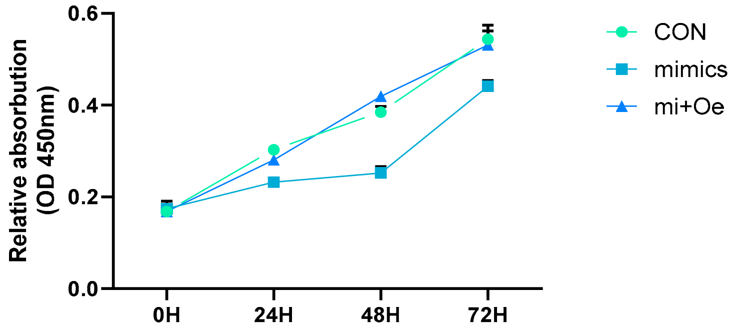

Supplementary Figure S11. FZD4 restoration rescues the inhibitory effect of miR-424-5p on osteosarcoma cell proliferation. Cell proliferation was assessed using the CCK-8 assay in the control (CON), miR-424-5p mimic (mimics), and miR-424-5p mimic plus FZD4 overexpression (mi+Oe-FZD4) groups at 0, 24, 48, and 72 h. Cell viability was determined by measuring absorbance at 450 nm. Data are presented as the mean ± SD.

Supplementary Figure S12

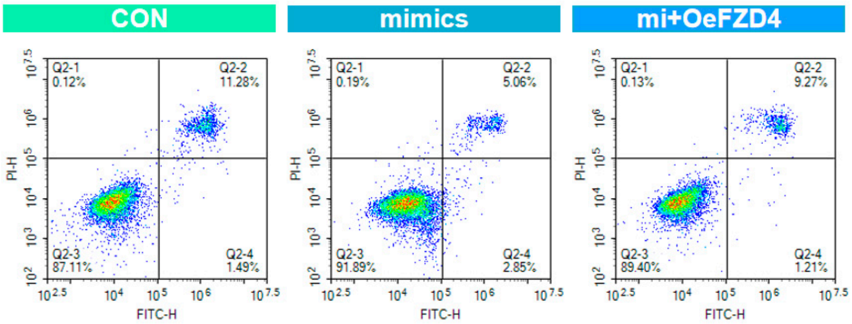

Supplementary Figure S12. FZD4 restoration rescues the inhibitory effect of miR-424-5p on the $CD133^{+}/CD44^{+}$ stemness-associated cell populations in osteosarcoma cells. Representative flow cytometry plots showing the distribution of stemness-associated cell populations in the control (CON), miR-424-5p mimic (mimics), and miR-424-5p mimic plus FZD4 overexpression (mi+Oe-FZD4) groups.

Supplementary Figure S13

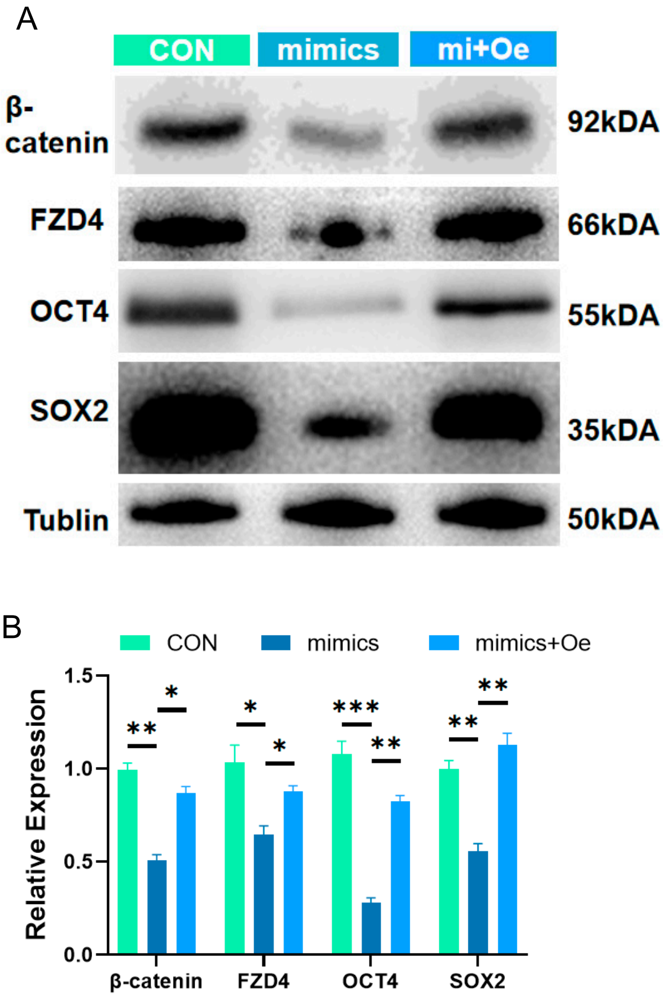

Supplementary Figure S13. FZD4 restoration reverses miR-424-5p-mediated suppression of β-catenin and stemness-associated proteins in osteosarcoma cells.

(A) Representative Western blot images showing the protein expression levels of β-catenin, FZD4, OCT4, and SOX2 in the control (CON), miR-424-5p mimic (mimics), and miR-424-5p mimic plus FZD4 overexpression (mimics+Oe-FZD4) groups (Image S7). (B) Quantitative analysis of the relative protein expression levels normalized to Tubulin. Data are presented as the mean ± SD. *$p < 0.05$, **$p < 0.01$, ***$p < 0.001$.

Supplementary Figure S14

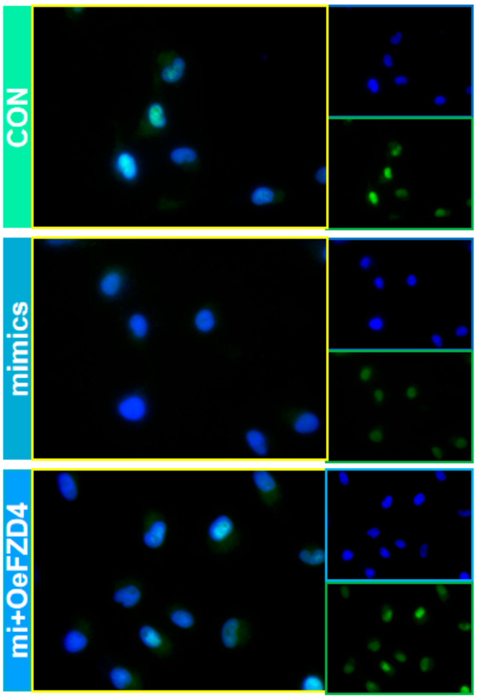

Supplementary Figure S14. FZD4 restoration reverses miR-424-5p-mediated suppression of β-catenin in osteosarcoma cells. Representative immunofluorescence images showing β-catenin expression in the control (CON), miR-424-5p mimic (mimics), and miR-424-5p mimic plus FZD4 overexpression (mi+Oe-FZD4) groups. β-catenin is shown in green, and nuclei were counterstained with DAPI (blue).

Supplementary Figure S15

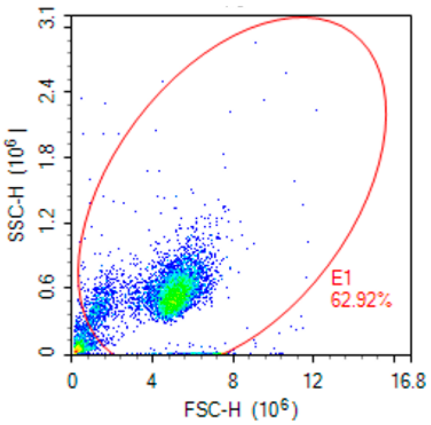

Supplementary Figure S15. Flow cytometry gating strategy. The main cell population was identified based on forward scatter (FSC-H) and side scatter (SSC-H) characteristics. The E1 gate was established to exclude cellular debris and non-target events, and approximately 62.92% of total events were included within the selected population for subsequent flow cytometric analysis.
